# Supplementary material for: Time to recovery from necrotizing enterocolitis and its predictors among neonates admitted to Neonatal Intensive Care Unit in Bahir Dar, Ethiopia: A retrospective follow up study, 2022
Source: PLoS One. 2024 Oct 22;19(10):e0311890. doi: 10.1371/journal.pone.0311890 (PMC11495620; doi:10.1371/journal.pone.0311890)
Supplement: Attachment — Submitted filename: Comments for Manuscript on NEC.docx [file pone.0311890.s002.docx]

**Title:** Time to Recovery from Necrotizing Enterocolites and Its Predictors among Hospitalized Neonates in Bahir Dar, Ethiopia: A Retrospective Follow up Study.

**Abstract:**

**Line 39-43**- All of these predictors have a weak strength of association, do you think, based on such strength your recommendation will be acceptable?

**Line 46-49:** How can assured that they were not give attention? Your recommendation better to be modified based on your findings, but not based on your real facts.

**Introduction:**

**General comment:** It didn’t show the gap that the findings of this research will fill. Previous works of literatures were not dig out well, poor literature review you did. The general write up was not good including the language, better to be modified. The coherence of the write was not good, better to be revised. Finally, better to precisely and coherently written with adequate literature review.

**Methods:**

**Line 160-64:** I think such type of sample size determination formula is not appropriate for time to event data. I am eager to hear your justification why you are using margin of error=3???

**Line 204:** Are they professionals working at the study site?

**Line 227:** What are the method used for assumption checking for independent variables and when we accept the variable for regression or at least by what method the variable should fulfil the assumption to use cox proportional Hazard regression?

**Line 235:** Could you think this graph showed the model was fitted? Better to see or clean your data again and again before fitting the model and adequacy checking.

**Result:**

**Line 259: Table 2:** In Your statement of justification early age of neonate is risk factors, but here there is less risk difference. How do you justify it? Where did you get the age of the mother, hence it is a chart review for neonates and if there is the chance to get maternal data, why not include maternal related other variables?

**Table 3:** What is the prevalence of preterm birth in Ethiopia? Have you appreciate your descriptive statistics here? You know that preterm neonates are more prone to develop NEC than others if so is it comparable?

**Line 283 and 287**: What is the cumulative survival probability? And Did the service provision given at these hospitals reduce the neonatal mortality due to NEC??? Why such large proportion of neonates were died after admission? Have got insights regarding such death?

**Line 307:** Why most of the neonate were died after admission??? The NEC or the poor service delivery system??? Better to incorporate in your discussion such peculiar findings.

**Figure 4:** Did the graph fulfil the assumption? Did this graph fulfil the assumption statistically? Better to include statistically methods of assumption checking in addition to graphical methods?

**Table 6:** PNA is Well Known factor for NEC. Are you sure not having PNA is a cause for NEC??? Needs detail discussion. Most of your statistically significant variables had weak strength of association. How can you interpreted it? Do you think based on this findings recommendation is acceptable?

**Discussion:**

**General comments**: your discussion is poor even though your findings are peculiar including your write up, coherence and interpretation and implication. **Revised your discussion.**

**Line 361:** If it reported incidence of survival, which was censored, not comparable with your findings since you are reported incidence proportion of recovery, which was event.

**Line 372:** Do you think sample size difference a justification for result difference? What does sampling distribution mean?
