# Supplementary material for: Time to recovery from necrotizing enterocolitis and its predictors among neonates admitted to Neonatal Intensive Care Unit in Bahir Dar, Ethiopia: A retrospective follow up study, 2022
Source: PLoS One. 2024 Oct 22;19(10):e0311890. doi: 10.1371/journal.pone.0311890 (PMC11495620; doi:10.1371/journal.pone.0311890)
Supplement: Attachment — Submitted filename: Manuscript on NEC Final.docx [file pone.0311890.s003.docx]

**Time to Recovery from Necrotizing Enterocolites and Its Predictors among Hospitalized Neonates in Bahir Dar, Ethiopia: A Retrospective Follow up Study, 2022**.

Birtukan Ayana Tefera^1^*^¶^, Abdurahman Mohammed Ahmed^2^*^¶^, Sisay Shewasinad Yehualashet^3^*^¶^

1. Neonatal intensive care unit, Felege Hiwot comprehensive specialized hospital , Bahir Dar, Amhara Regional state, Ethiopia,

2. School of Public Health, Asrat Woldeyes Health Science Campus, Debre Birhan University, Debre Birhan, Amhara Regional state, Ethiopia.

3. School of Nursing and Midwifery, Department of Paediatrics and Child Health Nursing, Asrat Woldeyes Health Science Campus, Debre Birhan University, Debre Birhan, Amhara Regional state, Ethiopia.

**^*^Corresponding author**

Email: [birtukana681@gmail.com](mailto:birtukana681@gmail.com)

^¶^These authors contributed equally to this work.

**Abstract**

**Background:** Necrotizing enterocolites (NEC) is one of the most common devastating, life-threatening, acquired gastrointestinal disorder in neonates. It is a serious disorder which commonly affecting preterm, very low birth weight and asphyxiated neonates. Moreover, large proportion of neonates with necrotizing enterocolites disorder are highly vulnerable to death. Therefore, this study was aimed to assess the time to recovery from necrotizing enterocolites and its determinants among hospitalized neonates in public health hospitals in Bahir Dar City, Ethiopia.

**Methods:** An institution-based retrospective follow-up study design was employed. A sample of 361 medical records of neonates with necrotizing enterocolites were selected by systematic random sampling technique. KoBo Collect v20213.4 was used to collect the data using a checklist. Median was calculated to estimate survival time. The life table was used to estimate cumulative survival probabilities after admission. Kaplan Meier survival curve and log-rank test were used to describe the survival function. The independent effects of covariates on recovery time were analysed using multivariable Cox-proportional hazard model. The data was analysed by stata 14.0 software package.

**Results:** The median time to recovery from necrotizing enterocolites among hospitalized neonates was 12 days (interquartile range: 10-14 days). Multivariable Cox-proportional hazard model revealed that Stage I of necrotizing entrocolites (Adjusted Hazard Ratio (AHR): 2.09, 95% confidence interval (CI) = 1.11– 3.94), Neonatal weight 1500gm-2499(AHR: 1.63, 95% CI: 1.05–2.53), having respiratory distress syndrome (AHR: 1.9, 95% CI: 1.19– 2.67), being exposed for perinatal asphyxia (AHR: 1.8, 95% CI: 1.24– 2.67) and level of platelet count greater than 150,000 (AHR: 1.62, 95% CI: 1.14– 2.32), were statistically significantly predicted the time to recovery from necrotizing entrocolites.

**Conclusion:** The median time to recovery from NEC was slightly lower. This was mainly predicted by neonatal birth weight, level of disease progression, physiological response and clinical conditions of the neonates. Therefore, Ministry of health have to work in improving the capacity of treatment centres. Neonatal health care professionals have to giving more attention for susceptible neonates, equipped for early diagnosis and providing proper treatment to prevent deaths of the neonates

**Keywords:** Time to recovery, necrotizing entrocolites, Neonate, Hospital, Ethiopia

# INTRODUCTION

Necrotizing enterocolites (NEC) is one of the most common devastating, life-threatening, acquired gastrointestinal disorder in neonates (1, 2). It is characterized by a sudden onset of non-specific symptoms such as abdominal distension and/or discomfort, intolerance to enteral feeds, bloody stools, apneas, bradycardia, and temperature instability (3).

Necrotizing enterocolites is a major cause of neonatal morbidity and mortality, and both in developed and developing countries (4). It has been identified as the most common serious gastrointestinal disorder affecting preterm, very low birth weight (VLBW) new-borns, asphyxiated neonates (5). The cause of necrotizing enterocolites is unknown, but it is thought to be multifactorial. Prematurity is the most common cause of gastrointestinal immaturity. Term infants with co-morbidities such as heart defects and asphyxia also affected by NEC(6).

The incidence of NEC is 6% to 10% in neonates with birth weight less than 1500 gm (7, 8). The median survival time for low birth weight neonates with NEC was 3 days .This means that the majority of low-birth-weight neonates with NEC died during the early neonatal period (9). An overall mortality rate of infants with confirmed NEC as Bell stage two or greater was 23.5 percent(4). Bell's classification is divided necrotizing enterocolites into three stages, which is used to choose treatment options (2, 10). Re -Initiation of enteral feeding with in 7days in stage 1and 2 cases of NEC and 14days in stage 3 is necessary to avoid prolonged Nothing per os (NPO) period of time and parenteral nutrition it can increase the chance of intestinal atrophy (11).

Necrotizing entrocolites can quickly progress to a fulminant state with intestinal necrosis and perforation within hours to days. It usually occurs 2-3 days after birth, with 90% of cases occurring during the first 10 days of life (12). Premature new-borns account for 90% of cases, with gestational age having an inverse relationship (3). Although it could be as high as 20% in neonates born with low birth weight. Moreover, because of the co-morbid diseases that frequently accompany immaturity, recovery from NEC may be difficult (13).

Patients who develop surgical NEC are at a significant risk for morbidity and mortality, with long-term complications including short bowel syndrome, growth failure, and neurodevelopmental impairment (14).

But, when NEC is detected and treated early, the prognosis for babies with the condition is frequently very good. The majority of infants recover completely and have no long-term health problems and feeding problem (15).

(4). Necrotizing enterocolites is an expensive condition that accounts for about 20% of Neonatal intensive Care Unit (NICU) costs each year. Survivors of necrotizing enterocolites who require surgery frequently spend more than 90 days in the NICU and among those most likely to stay more than 6 months (17).

Given the significance of NEC as one of the top causes of new-born death, the World Health Organization recently declared the need to minimize maternal and neonatal morbidity as one of the Sustainable Development Goals and accepted a resolution to promote prevention, recognition, and care (18).

Prolonged parenteral nutrition administration for the treatment of NEC is linked to complications including sepsis and oedema due to fluid over load, and late re-feeding is linked to a longer hospital stay, Increased hospitalization length places a tremendous burden on the hospitalized neonate , his or her family, and health services especially in areas with poor resources and without health insurance which results increased medical costs or the need for additional services and potentially worse long-term neurodevelopmental outcomes are all linked to neonates with NEC. Surviving neonates, about one-fourth of all new-borns, experience substantial neurological effects as a result of central nerves system involvement (19).

According to the studies conducted Western world, the median recovery time after the onset NEC is different: in United states of America (USA) 10-14 days because any injury to the mucosa of the intestine requires at least 7 to 10 days (20), Netherland 14 days (21), and initiation of intestinal recovery occurs within 2 to 4 days after NEC onset (24) Also

Perevius study findings also identifies determinates of time to recover from NEC, which includes Gestational age, sex, neonates age at diagnosis, Stage of NEC (21), Citrlulen level . Birth weight associated with NEC survival NEC STGE associated with NEC survival (21), platelet count (28) Comorbid disorders, reducing transfusion and assuring early colostrum feeding improve outcome of neonate from NEC(9, 27).

Surgical management also have acquired the challenge of treating neonates who have NEC (23).

Neonates who had low Apgar score, breathing failure or resuscitated, CPAP ventilation ,fed mixed milk more likely to have a NEC and aggravate the disease severity it affect recovery when compared to neonates who had not faced those conditions (21).

Starting enteral feeds <7 days after a NEC diagnosis (29).and probiotic consumption (33) reduce the risk of developing medical complications associated with NEC including the recovery. Monitoring of gastric residual decreased age at full enteral feeding and Slow feeds advancement delayed after necrotizing entrocolites prolong the time of reaching full enteral feeding and increase the risk of invasive infection (30-32). Therefore, this study was determined the median time to recovery from necrotizing entrocolites as well as the identified predictors of time to recovery from necrotizing entrocolites in hospitals at Bahir Dar City, Ethiopia. This study finding is an input to policy makers, program managers and health professionals as an evidence to support decision making on neonatal health care programs.

# Methods and materials

## Study design and settings

An institutional based retrospective follow up study was employed in Public Hospitals at Bahir Dar City, Amhara region state, Ethiopia. Bahir Dar is the capital city of Amhara regional state and is found 563km northwest of Addis Ababa, which is the capital city of Ethiopia. There are three public Hospitals in the city, which are Felege Hiwot comprehensive specialized hospital, Tibebe Gion specialized Teaching hospital (TGSTH) and Addisalem hospital (AH). Currently the hospitals deliver health service as outpatient and inpatient management in different departments including neonatal intensive care unit services. All of them have neonatal specialists and neonatal nurses to care for neonatal with neonatal intensive care unit services equipment and facilities. Dataextraction was done from June to July 2022, which was amongneonates who admitted and got care at the three hospitals in the period from May 2019 to May 2022.

**Sample size determination and procedure**

Sample size (n) was determined using single population mean formula. Considering the following assumption: standard normal distribution with confidence interval (CI) of 95 %( Z=1.96), 10% contingency, tolerable margin of error (d=3) and standard deviation of 28 for time to reach a full enteral feeding (34). Based on those assumptions the calculated sample size was 369.

The study participants were selected from the registration book. The medical records of neonates who were admitted with necrotizing entrocolites from May 2019 to May 2022 were selected. The total number of neonates with necrotizing entrocolites admitted in NICU of Tibebe Gion, Felege Hiwot and Addisalem were 326,410 and 116 respectively, with a total number of 852 cases over the past 3 years. The sample sizes for each hospital were assigned proportionally based on their previous three years admission report of each hospital, which was 141 at Tibebe Gion hospital , 178 at Felege Hiwot hospital , and 50 at Addisalem hospital. Then, medical records of the neonates was arranged as per the registration book numbers sequence in each hospital in the specified time period. Finally, systematic random sampling technique with every 2 interval was applied to select medical record for data extraction after the first document was selected by lottery method. Incomplete charts for major variables (date of diagnosis, date of reaching full enteral feeding) were excluded from the study.

### Measurements

Necrotizing entrocolites was defined as an acute disorder of the gastrointestinal in the neonatal period who presented with clinical signs or radiographic findings of NEC and who diagnosed by attending physician. The primary outcome of this study finding was time to recovery from necrotizing entrocolites, which was determined by the time when the neonates who reach full calorie feeding which is 150 to 180 ml per kg per day. For the survival analysis, events were neonates who recovered from NEC during the follow up time, which was time from admission to the 14 days of life after diagnosis of NEC. Those neonates who referred, died, self-discharged for any reason before reaching full calorie feeding are censored. The s**urvival time was** the time starting from the date of admission to recovery from NEC. Birth weight of the neonates was classified as very low birth weight when less than 1500 gm, low birth weight 1500-2499 gm, normal birth weight 2500-3999 gm, and macrosomia when greater than 4000 gm (35). Early-onset of NEC was defined as the occurrence of NEC during the 1st week of life (≤ 7 days) and late-onset of NEC was its occurrence at any time after day 7 of life of the neonates (36). Gestational age was categorized **as** very preterm 28-31 weeks, moderate preterm 32-37 weeks, term when greater than 37 +1 weeks pregnancy (35). A platelet count of less than 150,000/microL defined as Thrombocytopenia (37, 38). Apgar **(Activity, Pulse, Grimace, Appearance and Respiration)** is a quick assessment performed on a baby at 1, 5 and 10^th^ minutes after birth and it has Three levels of score: Low APGAR score 0-3 - Moderate APGAR score 4-6 - Normal APGAR score 7-10.

The data was collected by Kobo Collect from neonate’s medical record using pre-tested data abstraction form, which prepared by reviewing different literatures. The checklist was containing three parts socio-demographic characteristics, neonatal characteristics and management option used for treating neonatal with NEC disorder.

### Data quality control

The data was collected by three BSc neonatal nurses. The data collectors were trained about the purpose of the study, confidentiality and data collection techniques as well as how to fill the checklist properly using the Kobo Collect from. Pre-test was done on 5% of the sample size. The investigators was supervised and checked the filled checklist for its completeness and consistency on daily basis.

## Data processing and analysis

The collected data using KoBo Collect v 20213.4 was exported to STATA 14.0 and checked for completeness and consistencies then cleaned and coded before actual data analysis. The data primarily organized and summarized using the descriptive statistics to show the characteristics of study variables. Days were used as a time scale to calculate time to recovery. The outcome of each participant was dichotomized into censored or event (recovered). Median was calculated for survival time. Kaplan Meier survival curve was used to describe proportion of recovery over time after the diagnosis of necrotizing entrocolites. The life table was used to estimate cumulative survival probabilities after admission. Kaplan Meier survival curve and log-rank test were used to describe the survival function.

Cox proportional hazards regression model was used to identify determinants of recovery from necrotizing enterocolites. All covariates with a p-value of 0.25 or less in the bivariate analysis were entered in to the multivariable model. Adjusted hazard ratios with 95% Confidence Interval (CI) was presented. A p-value less than 0.05 was a cut point to determine statistically significant association between recovery time and covariates. The proportional hazard assumption was checked by using global test and Schoenfeld residual test. The Multicollinearity was checked using Variance Inflation Factor (VIF) the mean result was VIF 1.92.

## Statistical model and assumptions

The overall adequacy of the final fitted model was checked by the Cox-Snell residuals were estimated based on the Kaplan–Meier estimated survivor function. This graphical plot of the cumulative hazard versus cox-Snell residuals showed an approximate straight line with slope one and indicates the model fits the data. And in addition to this, the final model was tested statistically for its fitness by using the global goodness of fit test which was decided as adequate if its p-value is greater than 0.05(figure 1).The global goodness of fit test of Cox proportional hazard assumption was checked for each covariate using Schoenfeld residuals test Based on this, final model was p-value = 0.4(Table 1).

Figure 1: Cox-Snell residual graph for the goodness of model fitness that shows the hazard function follows the 45° closed to the baseline, based on the Kaplan–Meier estimated survivor function, to test the overall adequacy of the Cox proportional hazard model of time to recovery from necrotizing entrocolites and its determinants among neonates who was admitted to NICU of FHCSH, AH and TGCSH, Amhara, Northwest Ethiopia

Table 1 : The global goodness of fit test of Cox proportional hazard assumption

|  | Chi2 | df | Prob>chi2 |
| --- | --- | --- | --- |
| global test | 25.35 | 25 | 0.4426 |

# Ethical consideration

Ethical clearance was obtained from Debre Berhan University Aserat Woldeyes Health Science Campus Institution Review Board. Support letter was written from Amhara public Health Institute (APHI) for each hospitals. Formal letter was submitted to FHCSH, TGSTH and AH and permission was assured. Permission was taken from quality improvement offices. All information collected from neonates’ cards was kept strictly confidential and anonymous.

# Results

## Socio-demographic characteristics of neonate and mother

A total of 369 hospitalized neonates medical record chart were reviewed in this retrospective follow-up study. Of them 8 charts were excluded because of incompleteness. Hence, a total of 361(97%) completed neonates medical record were included in this study. About 192(53%) were male and 269 (74.5%) were found at the age group of 1-7days, which early onset of NEC was occurred (Table 2).

Table 2: Socio-Demographic Characteristics of a neonate with necrotizing enterocolites Admitted from May 2019 to May 2022 at public hospitals in Bahir Dar.

| **Variables** | **Category** | **Frequency(Percentage)** | **Event** | **Censored** |
| --- | --- | --- | --- | --- |
| Age of the neonate(day) | 1-7 days | 258 (71.47%) | 163 | 95 |
|  | >7days | 103 (28.53%) | 60 | 43 |
| Age of the mother(year) | 18-34 | 301 (83.38%) | 188 | 113 |
|  | 35-45 | 60 (16.62%) | 35 | 25 |
| Sex | Male | 192(53%) | 119 | 73 |
|  | Female | 169(47%) | 104 | 65 |
| Place of delivery | Institutional | 355 (98%) | 220 | 135 |
|  | Home | 6 (2%) | 3 | 3 |

## Neonatal characteristics

The median weight was 1580 grams with (IQR 1350-2530). A minimum of 850 grams to a maximum of 4110 grams. Median gestational age was 34 wks (IQR 31-37wks). Among the participants of this study 342 (94.7%) of neonates Apgar score of 5^th^ minutes was greater than 5 and its mean was 7.65 with (SD ±1) (Table 3).

Table 3. Characteristics of neonate with necrotizing entrocolites who was admitted from May 2019 to May 2022 at public hospitals in Bahir Dar.

|  | | | | **Survival Status** | |
| --- | --- | --- | --- | --- | --- |
| **Variables** | **Category** | **Frequency(Percentage)** | | **Recovery** | **Censored** |
| Birth weight | < 1500gm | 149 | (41.3%) | 50 | 99 |
|  | 1500-2499gm | 114 | (31.6%) | 90 | 24 |
|  | 2500-3999gm | 94 | (26%) | 79 | 15 |
|  | >4000gm | 4 | (1.1%) | 4 | - |
| APGAR score in 5^th^ min | <5 | 19 | (5.3%) | 10 | 9 |
|  | >5 | 342 | (94.7%) | 213 | 129 |
| Stage of NEC | Stage 1 | 65 | (18%) | 60 | 5 |
|  | Stage 2 | 207 | (57.3%) | 149 | 58 |
|  | Stage 3 | 89 | (25.7%) | 14 | 75 |
| Gestational age | 29-31wk | 91 | (25.2 %) | 29 | 62 |
|  | 32-37wk | 182 | (50.4%) | 130 | 52 |
|  | >37wk | 88 | (24.4%) | 64 | 24 |
| Birth Type | Single | 288 | (79.8%) | 193 | 95 |
|  | Twin | 67 | (19.6%) | 30 | 37 |
|  | Triplet | 6 | (1.7%) |  | 6 |
| Platelet count | < 150,000 | 151 | (41.8%) | 41 | 110 |
|  | >150,000 | 210 | (58.2%) | 182 | 28 |
| RDS | Yes | 155 | (42.9%) | 37 | 118 |
|  | No | 206 | (57.1%) | 186 | 20 |
| PNA | Yes | 105 | (29.1%) | 36 | 69 |
|  | No | 256 | (70.9.4%) | 187 | 69 |
| MAS | Yes | 31 | (8.6%) | 18 | 13 |
|  | No | 330 | (91.4%) | 205 | 125 |
| Jaundice | Yes | 61 | (16.9%) | 34 | 27 |
|  | No | 300 | (83.1%) | 111 | 189 |

## Therapeutic management options used to treat the neonates

Of all neonates who were treated in NICU, 303 (83.9%) were feeding breast milk, and 351 (97.23%) were treated by medical management. The neonates were treated with two or more combined intravenous antibiotic, which includes ceftriaxone, ampicillin, gentamicin and metronidazole based on the severity of disease. Most neonates in respiratory failure were assisted by respiratory support, 132 (36.6%) were assisted by INO2 and 131 (36.3%) by CPAP (Table 4).

Table 4. Therapeutic management options used to recovery the neonates with necrotizing entrocolites among neonates admitted from May 2019 to May 2022 at public hospitals in Bahir Dar, in 2022.

|  | | | | **Survival Status** | | | |  |
| --- | --- | --- | --- | --- | --- | --- | --- | --- |
| **Variables** | **Category** | **Frequency (%)** | | **Recovery** | | | **Censored** |  |
| Type of feeding | Brest milk | 303 | (83.9 %) | | 189 | 114 | | |
|  | Formula | 44 | (12.2%) | | 26 | 18 | | |
|  | Mixed | 14 | (3.9%) | | 8 | 6 | | |
| Type of management | Medical | 351 | (97.23%) | | 220 | 131 | | |
|  | Surgical | 10 | (2.8%) | | 3 | 7 | | |
| Type of oxygen support | INO2 | 132 | (36.6% ) | | 99 | 33 | | |
|  | CPAP | 131 | (36.3% ) | | 41 | 90 | | |
|  | Off | 98 | (27.1% ) | | 83 | 15 | | |
| Gastric residual monitoring | Yes | 187 | (51.8% ) | | 117 | 70 | | |
|  | No | 174 | (48.2% ) | | 106 | 68 | | |
| Feeding advancement | 15-20ml | 272 | (75.3% ) | | 172 | 100 | | |
|  | 30-40ml | 89 | (24.7%) | | 51 | 38 | | |
| Blood transfusion | Yes | 80 | (22.2%) | | 32 | 48 | | |
|  | No | 281 | (77.8%) | | 191 | 90 | | |

## Survival status and Incidence of neonates on time to Recovery

A total of 361 neonates were followed for 3667 person-days of risk time. The median follow-up time was 10 days with the minimum and maximum follow-up day of 2 and 14 respectively. The median survival time was 12 (IQR: 10–14) days. The cumulative probabilities of recovery starting at 4 days (99.7%) with (CI: 0.9799 - 0.9996). The overall incidence of recovery time was 6 per 100 (95% CI: 5.3, 6.9) person-days. Out of 361 neonates, 223 (61.8%) of them were recovered, 138 (38.2%) neonates with NEC were censored, from those 124 (34.3%) neonates died and 10 (2.8%) neonates had left against medical advice and 4 (1.1%) was disappeared.

The incidence rate that necrotizing entrocolites neonates recovery was 0.29, 0.7,1.1,1.45,2.03,2.34,2.85,2.98,3.1,3.09 and 1.82 per 100 person-day in the first 4, 5, 6, 7,8,9,10,11,10,12,13 and 14 days, respectively(Table 5).

Table 5. Over all life table of survival function

| Time | Beg. total | Recovery | lost | Survival | Standard error | 95% Conf. Int. |
| --- | --- | --- | --- | --- | --- | --- |
| 2 | 361 | 0 | 2 | 1.0000 | - | - |
| 3 | 359 | 0 | 6 | 1.0000 | - | - |
| 4 | 353 | 1 | 6 | 0.9972 | 0.0028 | 0.9801 0.9996 |
| 5 | 346 | 6 | 16 | 0.9799 | 0.0075 | 0.9583 0.9904 |
| 6 | 324 | 7 | 9 | 0.9587 | 0.0108 | 0.9312 0.9753 |
| 7 | 308 | 10 | 12 | 0.9276 | 0.0143 | 0.8938 0.9509 |
| 8 | 286 | 23 | 15 | 0.8530 | 0.0199 | 0.8091 0.8875 |
| 9 | 248 | 17 | 12 | 0.7945 | 0.0230 | 0.7450 0.8355 |
| 10 | 219 | 39 | 18 | 0.6530 | 0.0279 | 0.5953 0.7046 |
| 11 | 162 | 14 | 6 | 0.5966 | 0.0293 | 0.5367 0.6514 |
| 12 | 142 | 26 | 5 | 0.4874 | 0.0308 | 0.4258 0.5461 |
| 13 | 111 | 19 | 5 | 0.4039 | 0.0309 | 0.3432 0.4638 |
| 14 | 87 | 61 | 26 | 0.1207 | 0.0219 | 0.0820 0.1674 |

The overall Kaplan Meire estimates identified that the probability survival of neonates admitted with NEC was high in the first day of admission, which relatively decrease as follow up time increase. The overall median survival time of admitted neonates with NEC in this study was 12 (IQR 10-14) days. During the fourth day of hospital stay, 99.71 % of survival probability was observed with standard error of 0.0029 (95%CI :0.9799	0.9996). At the half of the study follow up day the probability of survival was also found to be 85%with standard error of 0.0199(95%CI: 0.80, 0.88). The overall study follow up of the survival probability was also found to be 12% with standard error of 0.0219 (95%CI: 0.0820	0.1674) (figure 2).

Figure 2 : Cumulative survival distribution of neonates with necrotizing entrocolites admitted from 2019 to 2022 at Bahir Dar Public Hospitals.

### Determinants of Time to Recovery from Necrotizing entrocolites

In a bi-variable Cox regression analysis, determinants such as HMD, PNA, RDS, GA, weight of the neonate, stage of NEC, type respiratory support, feeding re-initiation time and platelet count of the neonate at admission were associated with time to recovery.

After analysis of multivariable Cox regression, the variables PNA, RDS, weight of the neonate, stage of NEC, and platelet count were found to be significant determinants of time to recovery from necrotizing entrocolites.

The proportional hazard assumption was satisfied for the significantly associated variables. Neonates with the weight of 1500-2499gm 1.6 times (AHR: 1.63, 95% CI: 1.05–2.53) more likely to recover than neonates birth weight <1500gm. Neonates without RDS were 1.9 times (AHR: 1.9, 95% CI: 1.19–3.16) more likely to recover from NEC as compared counter parts (Figure 3).

Figure 3 :- Kaplan–Meier analysis for the association between time to recovery and respiratory distress syndrome (RDS) among neonates admitted in NICU.

A neonate without PNA were 1.8 times (AHR: 1.8, 95% CI: 1.24–2.67) more likely to recover from NEC compared as neonates who were diagnosed for PNA (Figure 4).

Figure 4 :- Kaplan–Meier analysis for the association between time to recovery and neonates diagnosed for perinatal asphyxia in NICU.

Those neonates were platelet count was >=150,000 1.6 times (AHR: 1.62, 95% CI: 1.14–2.32) more likely to recover than neonates platelet count <150,000 (Figure 5).

Figure 5 :-Kaplan–Meier analysis for the association between time to recovery and platelet count for neonates admitted in NICU.

Neonates who were diagnosed as stage I and stage II NEC were 2 times (AHR: 2.09, 95% CI: 1.11–3.94) and 1.9 times (AHR: 1.96, 95% CI: 1.09–3.51) more likely to recover than neonates who were diagnosed as stage III NEC respectively (Figure 6).

Figure 6:- Kaplan–Meier analysis for the association between time to recovery and necrotizing entrocolites stage for neonates admitted in NICU.

Table 6; Multivariable Cox Proportional Regression Analysis of determinant Factors for time to recovery from necrotizing entrocolites among neonates Admitted at Bahir Dar Public Hospitals, Northwest Ethiopia from 2019 to 2022.

| Variable | | Recovery | Censored | CHR (95%) | AHR (95% ) | P-value |
| --- | --- | --- | --- | --- | --- | --- |
| HMD | Yes | 142 | 91 | 1 | 1 |  |
|  | No | 81 | 47 | 1.47(1.11,1.93) | 0.86(0.62,1.18) | 0.360 |
| PNA | Yes | 36 | 69 | 1 | 1 |  |
|  | No | 187 | 69 | 1.69(1.18,2.42) | 1.82(1.24,2.67) | 0.002* |
| Reinitiating day of feeding | <5 | 192 | 109 | 1.54(1.05,2.25) | 1.29(0.86,1.92) | 0.208 |
|  | >5 | 31 | 29 | 2 | 2 |  |
| RDS | Yes | 37 | 118 | 1 | 1 |  |
|  | No | 186 | 20 | 3.16(2.22,4.50) | 1.94 (1.19,3.16) | 0.007* |
| Platelet count | <150,000 | 41 | 110 | 1 | 1 |  |
|  | >150,000 | 182 | 28 | 2.19(1.56,3.08) | 1.62(1.14,2.32) | 0.007* |
| Stage of NEC | Stage I | 60 | 5 | 4.64(2.59,8.30) | 2.09(1.11,3.94) | 0.021* |
|  | Stage II | 149 | 58 | 3.54(2.04,6.12) | 1.96(1.09,3.51) | 0.023* |
|  | Stage III | 14 | 75 | 1 | 1 |  |
| Weight of neonates | <1500gm | 50 | 99 | 1 | 1 |  |
|  | 1500-2499 | 90 | 24 | 2.4(1.7,3.39) | 1.63(1.05,2.53) | 0.028* |
|  | >4000 | 4 | 0 | 1.68(0.60,4.65) | 1.00(0.31,3.20) | 0.996 |
|  | 2500-3999 | 79 | 15 | 2.45(1.72,3.49) | 1.37(0.79,2.37) | 0.261 |
| GA | 29-31wk | 29 | 62 | 1 | 1 |  |
|  | 32-37wk | 130 | 52 | 1.79(1.20,2.69) | .81(0.48,1.38) | 0.452 |
|  | >37 | 64 | 24 | 2.51(1.61,3.89) | 1.2(0.64,2.46) | 0.493 |
| Respiratory support | CPAP | 41 | 90 | 1 | 1 |  |
|  | INO2 | 99 | 33 | 1.73(1.20,2.49) | 1.15(0.78,1.70) | 0.461 |
|  | Room air | 83 | 15 | 1.79(1.23,2.61) | .94(0.62,1.42) | 0.801 |

# Discussion

This study aimed to estimate the time to recovery from necrotizing entrocolites and its determinant factors among neonates admitted in the study hospitals within the study period. The overall incidence of recovery was 6 per 100 person-day of risk time. At the end of follow-up, 61.9% of neonates were recovered from necrotizing entrocolites. This finding is higher than the study done in Indonesia it shows incidence of survival was 44.2% (21). The difference in the amount of sample size they use and time when the studies were conducted may explain the discrepancy among study. Also the difference in comorbidity status among children could explain the variation in the finding.

This study revealed that the median recovery time from NEC was 12 days with (IQR 10-14). This is in line with studies conducted in Netherlands which indicated as the Median full enteral feeding time (FEFt) was 14 days and in U.S medical management course is usually 10 to14 days (21, 23).

In this study the median TTR from necrotizing entrocolites of the was 12 days (IQR 10-14) days respectively. This is smaller than the study done in Netherlands Median TTR after NEC was 20 (IQR: 16-30) days this difference might be due to the difference in study design and sample size (14).

This study outcome is higher than the previous study done in Los Angeles it may takes up to 5-7 days (39). This variation might be due to socio-economic differences in the study areas, time variation in which the studies were conducted. An additional possible reason for this discrepancy might be related to differences in treatment, care practices, and health care settings where the study was conducted. Another explanation for the discrepancy may be due to differences in baseline health conditions and treatment protocols.

At the multivariable level of analysis, the variables platelet count, birth weight, stage of NEC and comorbidities like PNA and RDS were significant predictors of time to recovery from NEC.

The platelet count of neonate was an important predictor in this research, and it had a major impact on the recovery of necrotizing entrocolites, This study indicated that neonates with a platelet count is greater than 150,000 were neonates recover earlier than those with platelet count less than 150,000 This finding is not consistent with study finding conducted in Indonesia (21). This discrepancy might be due to the difference in the result of variation in baseline clinical conditions. Another possible reason this may due to the difference in quality of services.

Other factor associated with recovery of necrotizing entrocolites was birth weight of the neonate, in this study neonates with the birth weight of 1500-2499gm were1.6 times (AHR: 1.63, 95% CI: 1.05–2.53) more likely to recover faster than neonates with birth weight less than 1500gm.The result was in consistent with the study done on Outcomes and Prognostic Factors for Survival of Neonates With Necrotizing Enterocolitis in Indonesia and Necrotizing enterocolites in low birth weight infants in China Mortality risk factors expressed by birth weight categories. Weight of the neonate was found to be a significant determinant of the recovery and a longer hospital stay in necrotizing entrocolites (21, 40).

The immaturity of intestine and increased susceptibility to intolerance, infection and complications could be a possible explanation for a prolonged time to recovery among very low birth weight neonates (21).

In this study, the stage of NEC was an important neonatal related predictor that has a significant effect on the recovery time of NEC patients it indicated that stage I and stage II neonates recover sooner than those with stage III. This finding is in line with the study conducted on Outcomes and Prognostic Factors for Survival of Neonates With Necrotizing Enterocolitis reported that NEC staging is a strong prognostic factor for the survival of neonates with NEC (21, 41, 42).

This might be when necrotizing enterocolites in stage I and stage II the hemodynamic abnormality and metabolic abnormalities are less severe than those neonates with stage III.

Another significant clinical indicator of TTR from necrotizing entrocolites was the presence of comorbidities like RDS and PNA. This suggests that neonates admitted with comorbidities of respiratory distress and perinatal asphyxia recovered lately than neonates who were admitted without those illnesses .This result is in line with the findings of a follow-up study conducted in USA(27).Comorbid disorders that typically accompany immaturity can make recovery from NEC difficult.

# Conclusion

This study assessed the median time to recovery from necrotizing entrocolites and potential determinant factors. The median time to recovery from NEC was 12 (IQR10-14)days. Weight of the neonate, platelet count, stage of NEC, and RDS, PNA were statistically significant predictors of recovery time from the disease.

# Funding

This research did not receive any specific grant from funding agencies in the public, commercial, or not-for-profit sectors.

# Authors' contributions

BAT ; Methodology, Investigation, statistical analysis, Software, Supervision, Validation, Visualization, Writing, Review, Editing, and developing the manuscript.

SSY; Methodology, Investigation, statistical analysis, Software, Supervision, Validation, Visualization, Writing, Review, Editing, and developing the manuscript.

AMA; Methodology, Investigation, statistical analysis, Software, Supervision, Validation, Visualization, Writing, Review, Editing, and developing the manuscript.

# Declaration of interest

The authors declare that they have no conflict of interest among Authors

# Data availability

Data, data collection forms, data extracted from included studies, data utilized for all analyses, and any other materials used in the review are all available from the corresponding authors.

# Acknowledgements

I would like to thank Debre Birhan University, Aserat Woldeyes Health Science Campus, and Department of Paediatrics and Child Health Nursing for giving us the chance to conduct this study. Also, we would like to express our gratitude for hospitals administrators, staffs and data collectors for their kind contribution in the data collection. We would like to acknowledge the authors of the included articles and Mr. Semahegn G for his English language spelling and grammar edition.

# References

1.	NEC IBNE. Necrotizing enterocolitis. MANUAL OF NEONATAL CARE. 2018:340.

2.	Battersby C, Santhalingam T, Costeloe K, Modi N. Incidence of neonatal necrotising enterocolitis in high-income countries: a systematic review. Archives of disease in childhood Fetal and neonatal edition. 2018;103(2):F182-f9.

3.	Author: Shelley C Springer J, MD, MSc, MBA, FAAP; Chief Editor: Muhammad Aslam, MD. Necrotizing Enterocolitis. Dec 27, 2017.

4.	Patel RM, Ferguson J, McElroy SJ, Khashu M, Caplan MS. Defining necrotizing enterocolitis: current difficulties and future opportunities. Pediatric research. 2020;88(1):10-5.

5.	Eaton S, Rees CM, Hall NJ. Current research on the epidemiology, pathogenesis, and management of necrotizing enterocolitis. Neonatology. 2017;111(4):423-30.

6.	Sharma R, Hudak ML. A clinical perspective of necrotizing enterocolitis: past, present, and future. Clin Perinatol. 2013;40(1):27-51.

7.	Bell MJ, Ternberg JL, Feigin RD, Keating JP, Marshall R, Barton L, et al. Neonatal necrotizing enterocolitis. Therapeutic decisions based upon clinical staging. Annals of surgery. 1978;187(1):1-7.

8.	Daniel R. Dirnberger M. necrotizing entrocolites. Necrotizing Enterocolitis Neonatology at Nemours Children's Health. october 2018.

9.	Kuik SJ. Necrotizing enterocolitis: Survival, intestinal recovery, and neurodevelopment. 2020.

10.	Christian VJ, Polzin E, Welak S. Nutrition management of necrotizing enterocolitis. Nutrition in Clinical Practice. 2018;33(4):476-82.

11.	Jones IH, Hall NJ. Contemporary outcomes for infants with necrotizing enterocolitis—a systematic review. The Journal of pediatrics. 2020;220:86-92. e3.

12.	Gephart SM, McGrath JM, Effken JA, Halpern MD. Necrotizing enterocolitis risk: state of the science. Advances in neonatal care : official journal of the National Association of Neonatal Nurses. 2012;12(2):77-87; quiz 8-9.

13.	Colleen Flahive M, Amy Schlegel,MD, Ethan A. Mezoff, MD,. Necrotizing Enterocolitis: Updates on Morbidity and Mortality Outcomes. The Journal of Pediatrics. January 16, 2020;Volume 220, P7-9, .

14.	Kuik SJ, den Heijer AE, Mebius MJ, Hulscher JBF, Bos AF, Kooi EMW. Time to full enteral feeding after necrotizing enterocolitis in preterm-born children is related to neurodevelopment at 2-3 years of age. Early Hum Dev. 2020;147:105091.

15.	Mekonnen SM, Bekele DM, Fenta FA, Wake AD. The Prevalence of Necrotizing Enterocolitis and Associated Factors Among Enteral Fed Preterm and Low Birth Weight Neonates Admitted in Selected Public Hospitals in Addis Ababa, Ethiopia: A Cross-sectional Study. 2021;8:2333794x211019695.

16.	Alsaied A, Islam N, Thalib L. Global incidence of necrotizing enterocolitis: a systematic review and meta-analysis. BMC pediatrics. 2020;20(1):1-15.

17.	Woelile TA, Kibret GT, Workie HM. Survival Status and Predictors of Mortality Among Low-Birth-Weight Neonates Admitted to the Neonatal Intensive Care Unit at Felege Hiwot Comprehensive Specialized Hospital, Bahir Dar, Ethiopia, 2020. 2021;12:451-66.

18.	The Global Maternal and Neonatal Sepsis Initiative: a call for collaboration and action by 2030. The Lancet Global health. 2017;5(4):e390-e1.

19.	Savarino G, Carta M, Cimador M, Corsello A, Giuffrè M, Schierz IAM, et al. Necrotizing enterocolitis in the preterm: newborns medical and nutritional Management in a Single-Center Study. Italian journal of pediatrics. 2021;47(1):226.

20.	Kuik SJ, Kalteren WS, Mebius MJ, Bos AF, Hulscher JB, Kooi EM. Predicting intestinal recovery after necrotizing enterocolitis in preterm infants. Pediatric Research. 2020;87(5):903-9.

21.	Siahaan E, Adriansyah W, Sasmita AP, Fauzi AR, Dwihantoro A, Gunadi. Outcomes and Prognostic Factors for Survival of Neonates With Necrotizing Enterocolitis. Frontiers in pediatrics. 2021;9:744504.

22.	David K. Stevenson MMLB, MD. Historical Perspectives: Necrotizing Enterocolitis: An Inherited Or Acquired Condition? March 01 2006.

23.	Dan Brennan M. Necrotizing Enterocolitis: Symptoms, Causes, Treatment. October 14, 2020

24.	Qian T, Zhang R, Zhu L, Shi P, Yang J, Yang CY, et al. Necrotizing enterocolitis in low birth weight infants in China: Mortality risk factors expressed by birth weight categories. Pediatrics and neonatology. 2017;58(6):509-15.

25.	Bora R, Murthy NB. In resource limited areas complete enteral feed in stable very low birth weight infants (1000-1500 g) started within 24 h of life can improve nutritional outcome. The journal of maternal-fetal & neonatal medicine : the official journal of the European Association of Perinatal Medicine, the Federation of Asia and Oceania Perinatal Societies, the International Society of Perinatal Obstet. 2017;30(21):2572-7.

26.	Markel TA, Engelstad H, Poindexter BB. Predicting disease severity of necrotizing enterocolitis: how to identify infants for future novel therapies. Journal of clinical neonatology. 2014;3(1):1-9.

27.	Cheryl A M, Susan Fullmer,Dennisl,Egget and RobertD. Factors affecting the sevierity of necrotizing entrocolites. Maternal -Fetal and Neonatal Medicine. May, 15,2013;26(17).
