# Supplementary material for: Time to recovery from necrotizing enterocolitis and its predictors among neonates admitted to Neonatal Intensive Care Unit in Bahir Dar, Ethiopia: A retrospective follow up study, 2022
Source: PLoS One. 2024 Oct 22;19(10):e0311890. doi: 10.1371/journal.pone.0311890 (PMC11495620; doi:10.1371/journal.pone.0311890)
Supplement: Attachment — Submitted filename: Response for reviwer PLOS ONE.docx [file pone.0311890.s004.docx]

**Dear Academic Editor!**

**PLOS ONE**

**Response to Reviewers**

We are pleased to resubmit for publication version of “**Time to Recovery from Necrotizing Enterocolites and Its Predictors among Hospitalized Neonates in Bahir Dar, Ethiopia: A Retrospective Follow up Study, 2022**.” for a review as original research in PLOS ONE.

The comments of the editor and the reviewers were highly insightful and enabled us to greatly improve the quality of our manuscript. Therefore, based on the editor’s and the reviewers’ concerns we have made extensive edition in our manuscript. Especially we have extensively edited the manuscript by a professional language editor (English-language Teacher (Simegnew) thoroughly edited the manuscript for language usage, spelling, and grammar) before submitting the revised version. We have addressed yours’ concerns in a point by point format.

We look forward to hearing from you at your earliest convenience.

Thank you for your consideration of this manuscript!

Kind regards,

**Sisay S.**

On behalf of authors

**Editor and Review Comments to the Authors**

Reviewer comments and Author response

**Response for the Reviewer #1 comments**

Reviewer #1: Thank you, authors, for bringing up this essential study issue in your scholarly work. The results are valuable for practice and have a bearing on public health. I do have some major and minor concerns, though.

**Major concern**

1. You determine the recovery of baby from NEC by reaching full calorie intake (150 ml/kg or 180 ml/kg) and limiting the follow-up period to 14 days. The standard duration of therapy for NEC ranges from 7 to 14 days and also in some cases like Surgical NEC, prematurity, Stage III PNA it may take longer than that to achieve complete feeding. Therefore, the majority of the baby may be ignored or recruited to censor, especially in stage II and stage III NEC babies. This could lead to an incorrect conclusion. I am not clear why do you want to restrict the follow-up date? **Response:** Thanks the reviewer for your major concern. As you explained we determined the recovery of babies from NEC by reaching full calorie intake (150 ml/kg or 180 ml/kg). But we were not limited the follow up period in our proposal. But 14 days of follow up period was determined after literature review on the title. Also as you mentioned we agree that on some cases like Surgical NEC, prematurity, Stage III PNA it may take longer than 14 days to achieve complete feeding. Of the 138 censored, 124 (89.9% of the censored) or 124 (34.3%) of the total study participants were died in the follow up period and 10 (7.2%) of centered or 10 (2.8%) of the total cases were resisted/against medical advice. The high mortality rate of neonates with NEC after hospital admission may related to the severity of the disease, quality/standard of care provided in the hospitals. Therefore, this could not lead to an incorrect conclusion. The study's objective was to ascertain whether NEC might recover within 14 days and how long it would take. The median recovery time was 12 days on average (10 to 14 days). From the total of 361 neonate with NEC, 223 (61.8%) were recovered within 14 days. Based on the study finding, fasten recovery will be achieved  by treating the predictors, which included Stage of NEC, low birth weight, respiratory distress syndrome, perinatal asphyxia, and decreased level of platelet count. It is crucial to comprehend the length of recovery and predictors in order to prevent nosocomial infection, lower expenses, and better manage resources. We will also indicate this as a limitation (line 391-400) and recommend the researchers use the finding as a basis line data to do prospective cohort studies that extend longer than 14 days.

**Minor comments**

1) You stated that recovery time is slightly lower in line 43 of your conclusion. What does "slightly lower" mean? And what criteria do you use to determine whether something is lower or higher? It is better to describe in faster or prolonged.

**Response:** Dear Reviewer, I appreciate your feedback and suggestions. According to your advice, line 43-44 has been changed to median recovery time rather than slower or faster because, according to biostatistics expert Professor Taddese Awoke consent, it is preferable to leave the decision to the scholar in order to avoid making false judgment.

2) If your finding implies faster recovery time, your recommendation may be better related with enhancing or strengthening the existing practice.

**Response:** the comment accepted and considered in the revised version of this manuscript. It is corrected from line 45-49 and as well at conclusion part.

3) Your paper's introduction section is lengthy. Please keep it brief and to the point by combining related results into a single paragraph.

**Response:** as per your comment the introduction section revised by removing redundancy and margining similar idea, so the word count reduced from 2773 to 743 in the revised manuscript (from line 52-96)

4) You claimed that respiratory assistance was used to help those with respiratory failure in Line 254. You ought to state Respiratory distress, in my opinion. Furthermore, What about resuscitation considering that around 30% of babies have PNA?

**Response:** Thank you for your concern, Neonatal resustation was take place at delivery room, but we consider respiratory support given at NICU with CPAP, INO2 and other means.

5) Line 262 median follow-up time was 10 days. Remove it because it doesn't convey any vital information. Instead, it is enough to describe the incidence density rate and median recovery time.

**Response:** Thank you, the comment accepted and it is removed, and the corrections take place from line 247-251

6) Be sure that the proportional hazard assumption is satisfied, unless using other parametric models is preferable. The PNA KM curve and NEC stage seem to contradict the assumption of hazard proportionality.

**Response:** Thank you for your notification, it was also our concern, but as to our consultation with a biostatisticians the PNA KM curve and NEC stage even it seems as it violating the assumption, it is not cross one group to the other comparison group so it satisfied the assumption.

7) Make the necessary corrections in Table 4 (Breast Milk), and in Table 6 (Reinitiating Day of Feeding Reference =1), Line 340 (Incidence Rate), Line 345 (Babies or Newborns),

**Response:** thank you for your comment and suggestion, all necessary correction was made as per your comment. Breast milk at table 4, Reinitiating Day of Feeding Reference =1 at table 6, incidence rate and newborns was corrected accordingly.

8) Please include some standards or scientific support for your discussion section beyond comparing your findings to those of others. Also, summarize comparable ideas or findings into a single paragraph.

**Response:** Thank you for your comment, by considered your comment, we reviewed the discussion section and necessary correction was taken from line 303- 384 as per the comment provided.

9) Avoid redundancy of ideas. For instance, lines 264 and 285; 271 and table 5; 299 and lines 360–362...

**Response:** Thank you, well accepted and correction was made in the revised version of our manuscript.

**Response for Reviewer #2 comments**

Dear reviewer thank you, your comment was sound and helpful for the improvement of this manuscript.

**Title:**

1) Time to Recovery from Necrotizing Enterocolites and Its Predictors among Hospitalized Neonates in Bahir Dar, Ethiopia: A Retrospective Follow up Study.

**Response:** thank you for your correction, the modification you made in the title is accepted and we modified on the revised version of our manuscript.

**Abstract:**

2) Line 39-43- All of these predictors have a weak strength of association, do you think, based on such strength your recommendation will be acceptable?

**Response:** Even if the correlation is not substantial, all of the factors found here are clinically or biologically feasible, and the 95% confidence intervals for each of them are small, indicating the accuracy of the estimate. In this regard, the recommendations may help to enhance the care given to newborns with NEC. Moreover, we recommended other researchers to conduct study using prospective cohort study design by including other variables not considered in this study. We included this weak association on the limitation of the study line 391-340

3) Line 46-49: How can assured that they were not give attention? Your recommendation better to be modified based on your findings, but not based on your real facts.

**Response:** The comment accepted and it is modified in the revised version of our manuscript from line 45-49 and on conclusion part. But we recommended like that based on looking to high mortality rate (34.4%) of the cases, which were related with the nature of the disease and severity of the problem, to mean there is a need to strengthening or enhancing the program which can reduce neonatal mortality after admission to hospitals.

**Introduction:**

4) General comment: It didn’t show the gap that the findings of this research will fill. Previous works of literatures were not dig out well, poor literature review you did. The general write up was not good including the language, better to be modified. The coherence of the write was not good, better to be revised. Finally, better to precisely and coherently written with adequate literature review.

**Response:** The comment accepted and the introduction section is modified and we tried to make it precise and coherent from line 52-96

**Methods:**

5) I think using single population formula, such type of sample size determination formula is not appropriate for time to event data.

**Response:** Dear reviewer thank you for your comment and suggestion. When we review different literature they used single population formula calculation by considering survival proportion for events. We communicated with biostatics professional and epidemiologist regarding to single population proportion formula, they said it is possible but it has its own limitation

6) Line 160-64: I think such type of sample size determination formula is not appropriate for time to event data. I am eager to hear your justification why you are using margin of error=3???

**Response:** Most researchers agree that a margin of error of 3% to 8% at the 95% confidence level is acceptable. The larger the margin of error, the less confidence one should have that a poll result would reflect the result of a census of the entire population. So, the margin of error increases as population variability increases; it decreases as population variability decreases. A smaller margin of error suggests that the survey's results will tend to be close to the correct values. Conversely, larger margin of error indicate that the survey's estimates can be further away from the population values. The margin of error is a measure of reliability. For a given confidence level, the smaller the margin error the more precisely we can pinpoint the true mean. What does a 3% error margin mean? The data was estimated with a 95% confidence level of accuracy within 3 percentage points. When we use such sample size calculation formula. Margin of error of (d=3) is used due to being conventionally agree/ think as it is neutral or not too conservative. Moreover, we used it to increase the sample size because the event occurs rarely.

7) Line 204: Are they professionals working at the study site?

**Response**: The data collectors were not professionals working at the study site. For this study experienced data collectors who are working in other sites were requited to minimize bias or systematic errors.

8) Line 227: What are the methods used for assumption checking for independent variables and when we accept the variable for regression or at least by what method the variable should fulfil the assumption to use cox proportional Hazard regression?

**Response:** Methods used for assumption checking of independent variables for regression includes in survival analysis, it is highly recommended to look at the Kaplan-Meier curves for all the categorical predictors. This will provide insight into the shape of the survival function for each group and give an idea of whether or not the groups are proportional (i.e. the survival functions are approximately parallel). We also consider the tests of equality across strata to explore whether or not to include the predictor in the final model. For the categorical variables we will use the **log-rank test** of equality across strata which are a non-parametric test.  For the continuous variables we will use a **univariate Cox proportional hazard** regression which is a semi-parametric model.  We will consider including the predictor if the test has a p-value of 0.2 – 0.25 or less.  We are using this elimination scheme because all the predictors in the data set are variables that could be relevant to the model. If the predictor has a p-value greater than 0.25 in a univariate analysis it is highly unlikely that it will contribute anything to a model which includes other predictors. It is stated on line 162-197

9) Line 235: Could you think this graph (figure 1) showed the model was fitted? Better to see or clean your data again and again before fitting the model and adequacy checking.

**Response:** We see that the hazard function follows the 45 degree line very closely except for very large values of time. It is very common for models with censored data to have some twisting at large values of time and it is not something which should cause much concern. Overall we would conclude that the final model fits the data very well, mean that the model was fitted

**Result:**

10) Line 259: Table 2: In Your statement of justification early age of neonate is risk factors, but here there is less risk difference. How do you justify it? Where did you get the age of the mother, hence it is a chart review for neonates and if there is the chance to get maternal data, why not include maternal related other variables?

**Response:** As it is stated in the introduction section, NEC usually occurs 2-3 days after birth with 90% of cases occurs during first 10 days of life. As we see from the frequency distribution 258 (71.5%) of the cases occurred in the first 7 days of the neonates (Table 2), for your information 333(92.2%) of the cases of NEC occurred during the first 10 days of life in this study. Concerning the age of the mother we got it from the neonatal chart during the review (registration book, including maternal age). Regarding, why not include maternal related other variables? When we do pre-test for the check list other variables, there were not complete in each neonatal chart, and then we removed from our checklist after the pre-test done.

11) Table 3: What is the prevalence of preterm birth in Ethiopia? Have you appreciate your descriptive statistics here? You know that preterm neonates are more prone to develop NEC than others if so are it comparable?

**Response**: Dear reviewer your comment is helpdful, the prevalence of preterm birth in Ethiopia is estimated around 10.48 (95% CI 7.98-12.99) as per the systematic review based on studies done from 2009-2019, which published in 2020 by BMC pregnancy and childbirth. But in this study more than 75% of neonates were less than or equal to 37 weeks. It was high because, this is due to the nature of the disease NEC most commonly affects the preterm neonates.

12) Line 283 and 287: What is the cumulative survival probability? And Did the service provision given at these hospitals reduce the neonatal mortality due to NEC??? Why such large proportion of neonates were died after admission? Have got insights regarding such death?

**Response:** cumulative survival probability is the set of probabilities used in estimating the probability of death or survival at each year and the cumulative probability of survival to each year is called a life table. Our way of explanation is completely not correct. So the way of interpretation changed like this “Of 361 neonates were followed the 1^st^ neonate was recovered at the 4th day of the follow-up”. As a researcher we believe that this much death after being admitted at the Hospital’s neonatal intensive care unit is not only due to disease severity but also due to quality of care provision or poor service delivery. That is why we recommended the ministry of health to give more attention in improving the quality of NICU services to reduce this much mortalities after hospital admission.

13) Line 307: Why most of the neonate were died after admission??? The NEC or the poor service delivery system??? Better to incorporate in your discussion such peculiar findings.

**Response:** This comment accepted and we incorporated this finding in the discussion section of this manuscript.

14) Figure 4: Did the graph fulfil the assumption? Did this graph fulfil the assumption statistically? Better to include statistically methods of assumption checking in addition to graphical methods?

**Response:**

**Kaplan-Meier statistic** allows researcher to estimate the survival rates based on three main aspects: survival tables, survival curves, and several statistical tests to compare survival curves. İn the most of the cases, researchers use the log-rank test without taking into consideration for assumptions. As stated on the methodology part of Statistical model and assumptions.

**The Kaplan-Meier plot** can be interpreted as follow: The horizontal axis (x-axis) represents time in days, and the vertical axis (y-axis) shows the probability of surviving or the proportion of people surviving (in our study the proportion of recovered in days). The lines represent recovery time curves of the two groups. A vertical drop in the curves indicates an event.

**The log-rank p value**: The significance level is set to 0.05 in most cases. If the calculated p-value is greater than 0.05, the null hypothesis is retained. Based on the available data, it is then assumed that both groups have the same distribution curve. If the p-value is less than 0.05, the null hypothesis is rejected.

15) Table 6: PNA is Well Known factor for NEC. Are you sure not having PNA is a cause for NEC??? Needs detail discussion. Most of your statistically significant variables had weak strength of association. How can you interpreted it? Do you think based on this findings recommendation is acceptable?

**Response:** you are right, PNA is well known factor for NEC. But we are not assessing the cause or predictors of NEC in this study; rather we are assessing predictors of NEC recovery time. Here the analysis identified that those neonates not having PNA were more likely to recover from NEC than those neonates who have PNA. As we given response above on the abstract section: Even if the strength of association not strong, all the variables identified here are clinically or biologically plausible and the 95%CI of all of them are narrow, which indicates preciseness of the estimate. In these sense the recommendations can work to improve the health care provision of the neonates with NEC. Moreover, we recommended other researchers or institution to conduct this research using prospective cohort study design by including other variables not considered in this study.

**Discussion:**

16) General comments: your discussion is poor even though your findings are peculiar including your write up, coherence and interpretation and implication. Revised your discussion.

**Response:** This comment well accepted and the discussion section revised accordingly from line 303-384

17) Line 361: If it reported incidence of survival, which was censored, not comparable with your findings since you are reported incidence proportion of recovery, which was event.

**Response:** It is overall survival, which is of all neonates with NEC who admitted to study site hospital and included in that study and survived (recovered) from NEC. So it is corrected accordingly.

18) Line 372: Do you think sample size difference a justification for result difference? What does sampling distribution mean?

**Response:** With understanding the concept of sampling distribution mean, which is a fundamental assumption to inferencing the finding from sample to the population from which the sample was drown. The concept of sampling distribution mean proofs the mean of sample mean distribution equals to population mean and the variance of sample mean distribution equals to population variance divided by the square root of the sample size used to form the sample mean distribution. Also, sampling distribution mean concept confirm that the smaller the sample size the more variability and less precis, which directly indicates sample size difference may cause differences in the estimated values even which is drown from the same population. For this discussion we preferred to state general facts rather than sample size difference.

Thank you again for your valuable comments and suggestion
