# Supplementary material for: Time to recovery from necrotizing enterocolitis and its predictors among neonates admitted to Neonatal Intensive Care Unit in Bahir Dar, Ethiopia: A retrospective follow up study, 2022
Source: PLoS One. 2024 Oct 22;19(10):e0311890. doi: 10.1371/journal.pone.0311890 (PMC11495620; doi:10.1371/journal.pone.0311890)
Supplement: Attachment — Submitted filename: Comment #Time to recovery#.docx [file pone.0311890.s005.docx]

***Time to Recovery from Necrotizing Entrocolites and Its Determinant Factors among Hospitalized Neonates in Bahir Dar, Ethiopia: A Retrospective Follow up Study, 2022***

Dear editorial team: Thank you very much for giving me the opportunity to review this manuscript

It is well known that NEC is one of the most common devastating, life-threatening, acquired gastrointestinal disorder in neonates. Despite this fact, why you are interested in studying time to recovery from NEC? Do you think that time is a big deal? Why you failed to deal about treatment outcome or survival status or something else?

From the abstract section: you should show the gaps and why you are going to deal about time to recovery from NEC?

The information presented from the system and the main document is different. So, which abstract is presented for review?

Reference 4 is missed

*Background:*

Long sentence with poor grammar typographical or grammatical errors should be corrected.

Most paragraphs are constructed with a single sentence and more complicated to understand. I suggest professional help in revising this manuscript. The flow of idea from one paragraph to the other paragraph is unrelated and difficult to understand.

Most of the idea presented in each paragraph is unrelated to the study objectives.

The overall incidence and /or prevalence of NEC is not stated clearly. The gap/why this study was carried out is not also described?

Data extraction period presented from the system and the main document is different. What is the reason for this discrepancy?

Why did you calculate sample size using single population mean rather than double population? In majority of health science studies we use tolerable margin of error (d=5%) but you use 3%. Could you explain your justification? The sample size calculation is still vague.

What is the source/study population?

What is the inclusion and exclusion criterion?

If the data was collected by neonatal nurse, how do you handle different types of bias? Why do you calculate median time instead of mean time to determine survival time?

Abbreviation/acronym should be written in full sentence during the first appearance. Eg FHCSH, TGSTH, AH, HMD, PNA, RDS, and GA

Discussion it needs major revision

First please remove statements described in the first two line of discussion section

When you write discussion, first you should discuss on the recovery time from NEC on similar studies conducted across the globe and put your scientific justification for the possible discrepancy if any then you can discuss on each explanatory variables starting from the local to global.

The statement presented on line number 346 is not similar/comparable with the idea stated on line number 347-49. So, it needs revision.

The statement described from line number 350-353 is not clear and is not presented well.

You are not expected to restate the results of the study rather comparing your study result with the other previously conducted researches and put the evidence for variations.

The possible reason raised during discussion is not sound and persuasive. So, it needs a major modification.

The discussion part is not written adequately and you should write starting from local to global unlike literature review from global to local.

In order to write a good discussions, you should search exhaustively similar studies done a broad and nationally. unless it becomes shallow.

You are also expected to discuss on the implications of the findings in context of existing research.

Based on your findings, you should write appropriate recommendation.

Limitation of the study is not written? Why
