# Supplementary material for: Time to recovery from necrotizing enterocolitis and its predictors among neonates admitted to Neonatal Intensive Care Unit in Bahir Dar, Ethiopia: A retrospective follow up study, 2022
Source: PLoS One. 2024 Oct 22;19(10):e0311890. doi: 10.1371/journal.pone.0311890 (PMC11495620; doi:10.1371/journal.pone.0311890)
Supplement: Attachment — Submitted filename: Response to Reviewers.docx [file pone.0311890.s006.docx]

Dear Editor and Reviewer,

Thank you for taking the time to review our article. We appreciate your valuable feedback and constructive comments. Your insights have provided us with a deeper understanding of the subject matter and have helped us improve the quality and clarity of our article.

Best regards,

**Response for the Reviewer #3 comments**

It is well known that NEC is one of the most common devastating, life-threatening, acquired gastrointestinal disorder in neonates. Despite this fact, why you are interested in studying time to recovery from NEC? Do you think that time is a big deal? Why you failed to deal about treatment outcome or survival status or something else?

1. Do you think that time is a big deal?

- **Response:** As you said NEC is one of the most common devastating, life-threatening, acquired gastrointestinal disorder in neonates. On this study the treatment outcome and survival status of NEC cases were presented in the result section. Also, Knowing the time to event (recovered or death/censed) in resource limited countries have importance to deal with the required resources in treating the NEC cases and giving insight for health care providers and concerned stakeholders on the required time to recover or survive from NEC among neonates. Intestinal recovery after NEC one of the most important predictors influence neonate survival, so timely recovery plays a crucial role in improving outcomes and reducing morbidity and mortality rates. So, one of the primary challenges in NEC management is early detection. Prompt diagnosis allows for timely initiation of treatment, which can significantly impact recovery time. However, the early signs and symptoms of NEC can be nonspecific and easily overlooked, leading to delayed diagnosis and intervention.

2. Why you failed to deal about treatment outcome or survival status or something else?

- **Response:** As it is mentioned above the treatment outcome and survival status of NEC cases were presented descriptively. But, predictors of treatment outcomes and survival status were not identified because the primary outcome of the study was to determine the time to recovery from necrotizing entrocolites and its predictor variables. On the current version of our manuscript in the result section on line 268-271 we mentioned that, “Out of 361 neonates, 223 (61.8%) of them were survived, 138 (38.2%) neonates with NEC were censored, from those 124 (34.3%) neonates died and 10 (2.8%) neonates had left against medical advice and 4 (1.1%) was disappeared without physician consultation. Concerning the treatment outcome, each type of treatment options in managing NEC cases with their treatment outcome (being survived or fail to survive) were presented in the result section on line 254-262 and summarized on (Table 4).

3. From the abstract section: you should show the gaps and why you are going to deal about time to recovery from NEC?

- Response: on line 21-23 the following sentence included, the time it takes for neonates to recover from NEC can vary widely, depending on the severity of the disease, the promptness of diagnosis, and the effectiveness of treatment interventions

4. The information presented from the system and the main document is different. So, which abstract is presented for review?

- Response: Thank you, I will submitted the same document on the system and the manuscript

5. Reference 4 is missed

- Response: Reference 4 was cited on line 56, in the introduction section, for the first time.

Background:

Long sentence with poor grammar typographical or grammatical errors should be corrected. Most paragraphs are constructed with a single sentence and more complicated to understand. I suggest professional help in revising this manuscript. The flow of idea from one paragraph to the other paragraph is unrelated and difficult to understand.

6. Most of the idea presented in each paragraph is unrelated to the study objectives.

- Response: thank you very much for your important comment. We acknowledge that English is not our first language and we have edited the manuscript by an English language instructor Ass/professor Simegnew (Debre Berhan University staffs). Based on your recommendations, we have made revision of the manuscript with yellow color accordingly.

7. The overall incidence and /or prevalence of NEC are not stated clearly. The gap/why this study was carried out is not also described?

- Response: thank you very much for your constructive comment that strengthens our manuscript: we incorporated prevalence and incidence of NEC data from line 91 -106.

8. Data extraction period presented from the system and the main document is different. What is the reason for this discrepancy?

- Response: Thank you, we will submitted the same document on the system and the manuscript

9. Why did you calculate sample size using single population mean rather than double population? In majority of health science studies we use tolerable margin of error (d=5%) but you use 3%. Could you explain your justification? The sample size calculation is still vague.

- **Response: Dear respected reviewer, it is known that** time to event data/study **has specific** sample size determination formula. But we can’t to get the required information to apply that formula during proposal preparation and assuming as time is a mean type of data we use single population mean formula to determine the sample size. Concerning using a tolerable margin of error (d=3), as you know it is possible to use a margin of error 1-5 for mean type of data or 1% - 5% for proportion type of data. But we used tolerable margin of error (d=3) due to being conventionally agree/ think as it is neutral or not too conservative. Moreover, we used it to increase the sample size and enhance the power of the study.

Furthermore, you are right, in majority of health science studies we use tolerable margin of error (d=5%) for proportion type of data. But as to my knowledge in advanced biostatistics course use of tolerable margin of error (d=5%) is correct and acceptable if the proportion of the study variable ranges from 0.4 to 0.6 (40% to 60%). Also, it is recommended to use tolerable margin of error (d=1%) if the proportion is less than 0.1 (10%) or proportion greater than 0.9 (90%), which enables as to get enough number of case or events.

10. What is the source/study population?

- **Response:** Source or target population is the total population interest that have something in common for which the information is required and we wish to draw conclusions/ generalizations at a particular time. Study population is the subset of the source/ target population from which a sample will be drawn or the specific population from which data are collected.
- In our study, “the source population was all neonates with necrotizing enterocolites who were admitted to neonatal intensive care unit of Hospitals in the study area. The study population was all neonates with necrotizing enterocolites who were admitted to neonatal intensive care unit of the study Hospitals during the study period, which was from May 2019 to May 2022.” This description of source and study population included on line number 133-137 in the methods section.

11. What is the inclusion and exclusion criterion?

- Response: Inclusion and exclusion criterion are conditions which indicates the eligibility criteria, which determine the members of the target population who can or can’t participate in the study.
- In our study, “All neonates with necrotizing entrocolites who were treated in neonatal intensive care unit of Felege Hiwot comprehensive specialized Hospital (FHCSH), Tibebe Gion Specialized Teaching Hospital (TGSTH) and Addisalem Hospital (AH) in period from May 2019 to May 2022 were included in the study. But all neonates with necrotizing entrocolites who were treated in neonatal intensive care unit of the three Hospitals and who had incomplete medical record or charts for major variables (date of diagnosis, date of reaching full enteral feeding) were excluded from the study.” This description of included criteria in the current version of our manuscript on line 137-139 in the methods section.

12. If the data was collected by neonatal nurse, how do you handle different types of bias? Why do you calculate median time instead of mean time to determine survival time?

- **Response:** We assumed that neonatal nurses were close to understand subject matter and had better practical experience on different medical chart which used at NICU, which enter enhance quality of the data. The data collectors were not professionals working at the study site. For this study experienced data collectors who are working in other sites were requited to minimize bias or systematic errors. Also, they are trained and the investigators undertaken re-check on randomly selected the filled data extraction form for its precision.

13. Abbreviation/acronym should be written in full sentence during the first appearance. Eg FHCSH, TGSTH, AH, PNA, RDS, and GA

- Response: Thank you for your comment, I try to correct accordingly on the manuscript part and highlighted with yellow colour: Felege Hiwot comprehensive specialized hospital (FHCSH), Tibebe Gion specialized Teaching hospital (TGSTH) , Addisalem hospital (AH), GA (Gestational age), RDS (Respiratory Distress Syndrome), PNA(Perinatal Asphyxia), MAS(Meconium Aspiration syndrome)

Discussion it needs major revision

14. First please remove statements described in the first two line of discussion section

- Response: Thank you for your comment, the first two lines is removed.
- We know that, it is important to include clear and comprehensive information in the discussion section in order to facilitate comparisons with other findings, but it is true that the absence of specific studies on recovery time can make the comparison challenging, We used the following strategies on the discussion part: We acknowledging that the lack of studies on recovery time on the limitation part to comparison this study with other findings. This demonstrates transparency and awareness of the research gap. We try to discuss the finding contextually thorough description of the methodologies, variables, and outcomes used in our study, so recovery time is not explicitly addressed. We discuss finding with related studies that are tangentially related to recovery time or explore similar concepts. This enables us to draw comparisons indirectly and highlight any parallels or discrepancies with the results. In the discussion part we consider broader implication of the finding beyond recovery time. We try to explore how the research finding contributes to the overall understanding of the topic, potentially shedding light on related factors that influence recovery or providing insights into other aspects of the condition or intervention being studied. On the conclusion part we suggest future research direction that could specifically investigate recovery time or address other gaps in the literature and connecting our finding with the existing body of knowledge, even if direct comparisons with other findings on recovery time are not possible.

15. The statement presented on line number 346 is not similar/comparable with the idea stated on line number 347-49. So, it needs revision.

- Response: Thank you it is corrected from line 362-364

16. The statement described from line number 350-353 is not clear and is not presented well.

- Response: Thank you it is corrected from line 371-379

17. You are not expected to restate the results of the study rather comparing your study result with the other previously conducted researches and put the evidence for variations.

- Response: thank you for your constructive comments: In this particular context, the absence of existing literature or research on the subject makes it challenging to draw upon established findings or expert opinions. So, we try to make our discussion from related or tangential information to formulate a justifiable perspective. And also we consider alternative sources of information, including anecdotal evidence, expert opinions, empirical observations, or analogous studies from related fields. We stated the gab on the limitations part of study. Although the absence of literature poses challenges, it should not discourage us from presenting a well-reasoned justification. By applying logical thinking, exploring alternative sources of information, and recognizing the need for further research, we can still provide a thoughtful and valid perspective on the subject matter at hand.

18. The possible reason raised during discussion is not sound and persuasive. So, it needs a major modification.

- Response: Based on your comment we made major modification on the discussion part from line 334-425

19. The discussion part is not written adequately and you should write starting from local to global unlike literature review from global to local.

- Response: Thank you for your guidance and it is corrected accordingly

20. In order to write a good discussions, you should search exhaustively similar studies done a broad and nationally. Unless it becomes shallow.

- Response: We conducted an extensive search of open-access journals and databases but have been unable to find a similar study. However, it's important to note that the absence of similar studies doesn't necessarily mean that our research is invalid but we used different discussion writing mechanism

21. You are also expected to discuss on the implications of the findings in context of existing research.

- Response: We incorporated the finding implication to practice and context of existing research, on the discussion part from line 334-425

22. Based on your findings, you should write appropriate recommendation.

- Response: Thank you; we incorporate recommendation from line 456-488

23. Limitation of the study is not written? Why

- **Response:** Response: Thank you; we already stated limitation from line 489-499
