# Supplementary material for: Time to recovery from necrotizing enterocolitis and its predictors among neonates admitted to Neonatal Intensive Care Unit in Bahir Dar, Ethiopia: A retrospective follow up study, 2022
Source: PLoS One. 2024 Oct 22;19(10):e0311890. doi: 10.1371/journal.pone.0311890 (PMC11495620; doi:10.1371/journal.pone.0311890)
Supplement: Attachment — Submitted filename: NEC Review Comments.docx [file pone.0311890.s007.docx]

**Title:** Time to Recovery from Necrotizing Enterocolites and Its Predictors among Hospitalized Neonates in Bahir Dar, Ethiopia: A Retrospective Follow up Study, 2022 (PONE-D-23-07935R2)

The authors tried to address an important topic of clinical and health concern, and presented relevant findings. However, the manuscript has several issues that need to be addressed.

**General Comment:**

- “enterocolites” to “enterocolitis” (revise this thourghout the whole manuscript file)**.**
- The manuscript had numerous typographic errors (punctuation, grammar, and spelling errors). There are alot of subject-verb agreement errors, numerous tense problems, fragment sentences, active-to-passive voice shifts, punctuation errors and so on. Hence, I advice the authors to use valid language editor tools or at least invite native language experts. Otherwise, it’s difficult to address each and every edition and typographic errors here as a reviewer. The whole document need to be exhaustively revised cautiously.
- The manuscript is too long. Please try to reduce several contents and shorten it as much as possible.

**Topic:**

- I suggest to replace the word “hospitalized” with the term “admitted to the neonatal ICU” in Bahir Dar. Hospitalized is a vague term and doesn’t specifically represent neonates admitted to the ICU but all who visited the hospital for any reason with or without admission.

**In the Abstract part**

- Line 1-9: The gap statement (why it is needed to investigate the time to recovery from NEC) should be clearly stated in the backround sub-heading of the abstract part. The authors focus on dealing with NEC itself rather than the timing of recovery.
- In the results sub-heading of the Abstract section, the identified predictors are not consistent. For example, RDS & PNA are expected to have negative effect and Platelet count and Stage I NEC are expected to predict recovery positively. Please try to make your findings and their interpretation consistent. Either use positive effects or negative effects by adjusting the reference category while running multivariable Cox-PH regression.
- Again in the conclusion section of Abstract, consistency rule is violated yet (e.g. NEC stage 1 vs advanced NEC, lower Plt count vs Plt>150,000...). You presented the findings positively in the result part but summaries were drawn negatively. Be consistent in the interpretation of your findings!
- “The median time to recovery from NEC was 12 days.” This statement could not be a summary/conclusion. It’s just a reptition of your findings. Conclusions should be interpretations of the findings rather. Please revise accordingly.
- “The ministry of health and other stakeholders should improve standard care practice...” Which standard care practice are you going to recommend to be improved? Please try to make it specific and in line with your findings during recommendation. None of your findings assessed and reported ‘standared care practice issues’; and thus, recommending this issue is not part of your objective.

**In the Introduction section:**

- The backround section focuses on describing the fatality and time to death of NEC as well as its devastating consequences on healthcare outcome and costs as well. This was nice. Thus, after reading the study background, readers expect to see in the same way from your study results. However, your ‘Title’ and your study findings contrarily focused on ‘time to recovery’ rather than ‘time to death and fatality’ (Lines 60-82). I suggest re-write either of the two parts (either the background or amend your topic and findings from results through discussion and conclusion).
- The authors focused on describing the burden of NEC exhaustively. Nevertheless, they failed to state/address the main problem/focus of the study, which was time to NEC rather than the magnitude or incidence of NEC. Thus, the whole introduction section requires thourough revision in this context.
- Lines 91-105: These statements can be merged with the 2nd paragraph of the introduction part since the incidence and magnitude are ways of describing diseases burden (morbidity and mortality). Avoid redundancies please!
- Line 109-118: “Even with early aggressive treatment, NEC still progresses and has significant morbidity with chronic gastrointestinal complications and poor neurodevelopment....”. Does it mean those adverse health outcomes related to the time of NEC recovery or the disease (NEC) itself? Rather you better state your gap statement specific to your topic of interest (time to NEC recovery) rather than NEC in general. For example, interms of longer recovery vs shorter recovery time after the diagnosis of NEC. Otherwise, your backround and statement will be suitable to study incidence of death/recovery from NEC itself rather than the timing (time of recovery). The two concepts are quite different! Please try to modify accordingly.

**In the Methods and materials section**

- Lines 121-22: “Institution-based retrospective follow-up study was conducted among NEC neonates in the early neonatal period...”. What does it mean early neonatal period? Does it mean you didn’t consider cases in the late neonatal period? If so you need to revise your population below and results as well. Anyways, be notified and make the required changes accordingly.
- Line 125: “An institution-based retrospective follow-up study was conducted…” avoid redundancy. Please consider deletion.
- Line 140-144: I have a major concern on the appropriateness of the formula that you have used to calculate the required sample size. First, since your study is a follow-up study, single population proportion formula cannot handle/consider follow-up groups equally in the sample estimation process, and thus, is not suitable for such type of studies. Conversely, there are appropriate and scientifically sound methods of sample size determination for survival studies including Cox-model and log-rank methods among others. Why don’t you use one of these methods? Secondly, you couldn’t use a variable that you didn’t assess for sample size estimation. In your case, you used “time to reach a full enteral feeding” for sample size calculation, and you miss it in your results part though. Please try to forward if you have convincing and statistically acceptable rationale for those points!
- Line 157-59: “Necrotizing enterocolites was defined as an acute disorder of the gastrointestinal in the neonatal period who presented with clinical signs or radiographic findings of NEC and who diagnosed by attending physician.” This definition is pure scientific definition and could not be considered as operational definition/measurement. Furthermore, how did you exactly diagnose NEC for the purpose of your study (Radiology findings, blood tests, clinically or what? What were the criteria used to declare whether the newborn had NEC or not? This should be clearly specified. Because sometimes cases which were diagnosed as NEC by the clinician/physicians in the charts might not fulfill the criteria of NEC diagnosis for your study (fase diagnosis) whereas some others might fulfill the criteria despite the clinicians missed it actually (unrecognized cases). Therefore, please revise this section as required, and otherwise it will still be a major concern that could endanger the validity of your findings! Finally, cite the appropriate reference for the definitions provided.
- Line 173-76: “Apgar (Activity, Pulse, Grimace, Appearance and Respiration) is a quick assessment performed on a baby at 1, 5 and 10th minutes after birth and it has three levels of score: Low APGAR score 0-3 - Moderate APGAR score 4-6 - Normal APGAR score 7-10.” Please cite the source.
- Line 197-98: “Kaplan Meier failure curve was used to estimate the time to 198 recovery during the follow-up.” Why you prefer to use the failure cureve? Time to recovery should be best expressed in the survival function rather than failure. So why you preferred the failure curve?
- Lines 209-210: “The Cox proportional hazard model assumption was checked for variables in the Log-log plot (graphically),...”. Log-log plot is not an assumption test, it is a model fitness check rather. Revise it please.
- Lines 209-217: I strongly advise you to remove this paragraph completely since it’s already described adequately under the ‘data processing and analysis’ section above. It is just a redundancy and avoid it except for the Cox-Snell graph!
- Lines 227-33: Could you include the reference number/research protocol number that you have received from the IRB of DBU AWHSCI here under the ‘ethical consideration section? Please state that wether the IRB waiver the study since it was retrospective study, otherwise it couldn’t be conducted from the scratch. Besides, please say something about how to ensure the confidentiality and authonomy of the data collected. What measures did you take?

**Results part:**

- Table 1-6: In all tables, please add the sample size (n=…) in the table caption and specify the abbreviations and the denotation of symbols (i.e *, **) under the table footnote.
- Table 4: What were the medical and surgical managements? it needs a description in the table footnote, and specify each medical management modality with their detailed weight of contribution (%) in the description above the table (Table 4).
- Lines 254-56: “Most neonates in respiratory failure were assisted by respiratory support, 132 (36.6%) were assisted by intranasal oxygen (INO2) and 131 (36.3%) by Continuous Positive Airway Pressure CPAP.” What does it mean respiratory support? It need to be clearly specified/defined because respiratory support can also be provided using CPAP. So, it’s a vague term.
- Line 263-65: “...minimum and maximum follow-up time of 4 and 14 days... Of 361 neonates were followed the 1st neonate was recovered at the 4th day of the follow-up.” did you just consider among those recovered or among the whole samples? What was the first neonate died or transferred or censored and at what day of follow-up? You better describe minimum and maximum follow-up time in this context rather. Because, records of censored cases were considered in the statistical estimations generally. Additionally, does it mean you exclude neonates above the age of 14 days? Why did you so if it is not just a finding?
- Lines 265-66: “The overall incidence rate of recovery was 6 per 100 (95% CI: 5.3, 6.9) person-days observation.” Why did you prefer to use a denominator of 100 for the incidence? I recomend to use a denominator of 1000 since this is the default figure. Nonetheless, if you are interested to provide the incidence per 100 person-time observations, you can convert the time scale to weeks and can estimate the incidence per 100 person-weeks rather. I recommend to moderate this way.
- Lines 271-73: Don’t present everything you found here. You better pick specific time (e.g. 1st, 7th, & 14th days incidence). This way will be more meaningful.
- Lines 279-81: “The overall Kaplan Meire estimates identified that the probability recovery of neonates admitted with NEC was zero in the first 3 days of follow up, which relatively increase as follow up time increase.” I doubt that you interpreted your finding correctly! If the probability of recovery was zero in the first 3 days, this implies all the samples are died or censored and they have no left with any chance of survival/recovery. Because, the survival probability could not be bounced back once it reaches zero. Conversely, failure probability (chance of mortality and fatality) will be zero at the beginning of the follow-up since everyone is alive that time, and it becomes increasing with time when deaths recorded. Hence, I recommend you to re-check your survival setting before you conduct any analysis and re-run the analyses so that you will get the correct results. To sum-up, this finding and the graph above are completely wrong and need to be amended accordingly.
- Please delete figures 3-6 or you can provide them at the end of the manuscript as a supplementary files. Log-rank tests are non-parametrics descriptive statistical methods that used to compare survival diferences among categories. But, you mixed-up it under the inferential outputs (regression outputs) which is semiparametric one. So, Log rank should be described in line with the Kaplan-Meier curve but not with the Cox-regression outputs.
- Table 6: Weight of the neonate contains a cell with zero record. Thus, it should not be incorporated to the Model (multivariable analysis) since it violates the test assumption (not the model assumption but test assumption). Either totally remove it or recategorize it and re-run the multivariable analysis. However, as it stands, the regression output is invalid and predictors identified cannot be acceptable.
- Again in Table 6: It is scientifically declared and well known that PNA, RDS and respiratory support are highly interlinked/correlated. If a neonates APGAR score is dropped below 7, it’s considered as asphyxiated and if asphyxiated you certainly suspect respiratory distress. In turn, if in respiratory distress, he/she will require respiratory support. Thereupon, you have to suspect effect modification and you don’t have to include these variables in a single model. At least you need to conduct stratified analysis (by clasifying your in two six stratum). After that, you can decide whether the association you got is statistically acceptable or invalid. Consequently, I suggest you to re-run multivariable analysis after excluding/recategorizing birth weight and removing at least two of the three inter-linked variables listed above. If you fail to do so, your finding will be completely invalid!

**In the Discussion Part**

- Line 333-425: Please cite the references used for the scientific justifications and explanations provided throughout the discussion section. You just cited references for comparisons, but missed to cite the justifications.
- Please try to make it short and precise as much as possible. As it stands, it is difficult to follow and convey the main points. It’s exhaustive and takes awy interest to read as well. Hence, I recommend to shrink it to a maximum of two pages.
- Finally, please try to admit the limitation of your study that need to be taken in to consideration while using and interpreting the findings.

**In the Conclusions:**

- Don’t directly repeat the results. In the conclusion, it’s expected that the major findings are interprated and summarized meaningfully and in clinically sound manner.
- Please delete the statements from Line 435-455. Totally erase these from the manuscript. The conclusion should be summarized in a single paragraph succinctly and neatly.
- Lines 456-499: Please erase all the statements from the manuscript.
- Please provide a single statement showing the directions that need to be taken into consider addressing the identified problems (succinct recommendation statement) under the conclusion section (as the last sentence of the conclusion).
