# Supplementary material for: Time to recovery from necrotizing enterocolitis and its predictors among neonates admitted to Neonatal Intensive Care Unit in Bahir Dar, Ethiopia: A retrospective follow up study, 2022
Source: PLoS One. 2024 Oct 22;19(10):e0311890. doi: 10.1371/journal.pone.0311890 (PMC11495620; doi:10.1371/journal.pone.0311890)
Supplement: Attachment — Submitted filename: Response to the reviewer .docx [file pone.0311890.s008.docx]

I am writing in response to the comments provided by the reviewer regarding our manuscript titled Time to Recovery from Necrotizing Enterocolitis and Its Predictors among Neonates Admitted to Neonatal Intensive Care Unit in Bahir Dar, Ethiopia: A Retrospective Follow up Study, 2022 (PONE-D-23-07935R2).

We appreciate the time and effort the reviewer has dedicated to reviewing our work, and we would like to thank you and the reviewer for the opportunity to revise and resubmit our manuscript. We have prepared a point-to-point response addressing all the comments and suggestions provided by the reviewer. The manuscript outlines the changes made in the revised manuscript, along with detailed explanations for each modification

**General Comment:**

1. “enterocolites” to “enterocolitis” (revise this thourghout the whole manuscript file)**.**

**Response:** Thank you for your valuable feedback. I have reviewed the comment and made the necessary revisions throughout the entire manuscript file. The term "enterocolites" has been corrected to "enterocolitis" wherever it appeared. Please let me know.

2. The manuscript had numerous typographic errors (punctuation, grammar, and spelling errors). There are alot of subject-verb agreement errors, numerous tense problems, fragment sentences, active-to-passive voice shifts, and punctuation errors and so on. Hence, I advice the authors to use valid language editor tools or at least invite native language experts. Otherwise, it’s difficult to address each and every edition and typographic errors here as a reviewer. The whole document needs to be exhaustively revised cautiously.

**Response:** We appreciate the reviewer's feedback regarding the English grammar edition of the manuscript. We understand the importance of ensuring the accuracy and clarity of the language used in the document. We apologize for any typographic errors, punctuation, grammar, and spelling mistakes that may have been present. To address these concerns, we enlisted the assistance of a qualified English instructor, Semehagn.G, from Debre Berhan University, who provided language editing services for the manuscript. However, we acknowledge that there may still be areas that require further attention. In light of the reviewer's recommendation, we will take additional measures to ensure the manuscript undergoes a thorough and cautious revision. We were consider using valid language editor tools and we were make every effort to address all the necessary editions and typographic errors to improve the manuscript's clarity and readability.

3. The manuscript is too long. Please try to reduce several contents and shorten it as much as possible.

**Response:** Thank you, as per your suggestion we tried to review and shorten the manuscript. By eliminating redundancies, reorganizing sections, and focusing on the most essential points, we make the manuscript more concise.

**Topic:**

4. I suggest to replace the word “hospitalized” with the term “admitted to the neonatal ICU” in Bahir Dar. Hospitalized is a vague term and doesn’t specifically represent neonates admitted to the ICU but all who visited the hospital for any reason with or without admission.

**Response:** Thank you very much, the topic modified as per the suggestion like this: “Time to Recovery from Necrotizing Enterocolitis and Its Predictors among Neonates Admitted to Neonatal Intensive Care Unit in Bahir Dar, Ethiopia: A Retrospective Follow up Study, 2022*”*

**In the Abstract part**

5. Line 1-9: The gap statement (why it is needed to investigate the time to recovery from NEC) should be clearly stated in the background sub-heading of the abstract part. The authors focus on dealing with NEC itself rather than the timing of recovery.

**Response:** Thank you, it is well accepted, on line 19-22 the following sentence included, the time it takes for neonates to recover from NEC can vary widely, depending on the severity of the disease, the promptness of diagnosis, and the effectiveness of treatment given for the patients.

6. In the results sub-heading of the Abstract section, the identified predictors are not consistent. For example, RDS & PNA are expected to have negative effect and Platelet count and Stage I NEC are expected to predict recovery positively. Please try to make your findings and their interpretation consistent. Either use positive effects or negative effects by adjusting the reference category while running multivariable Cox-PH regression.

**Response:** Thank you very much, the comment accepted and the reference category changed for PNA and stage of NEC. And the direction of the association indicated. As you explained, occurrence of PNA and stage III NEC status had negative effect on the time to recover from NEC. But platelet count greater than 150,000 and birth weight other than very low birth weight had positive effect on the time to recover from NEC. This is mentioned in the abstract and also the result section of the manuscript.

7. Again in the conclusion section of Abstract, consistency rule is violated yet (e.g. NEC stage 1 vs advanced NEC, lower Plt count vs Plt>150,000...). You presented the findings positively in the result part but summaries were drawn negatively. Be consistent in the interpretation of your findings!

**Response:** Well accepted and modified as per the comment. We have revised the conclusions to accurately reflect the positive or negative aspects of the results consistently.

8. “The median time to recovery from NEC was 12 days.” This statement could not be a summary/conclusion. It’s just a reptition of your findings. Conclusions should be interpretations of the findings rather. Please revise accordingly.

**Response**: Well accepted and modified as per the comment on line 35-39.

9. “The ministry of health and other stakeholders should improve standard care practice...” Which standard care practice are you going to recommend to be improved? Please try to make it specific and in line with your findings during recommendation. None of your findings assessed and reported ‘standared care practice issues’; and thus, recommending this issue is not part of your objective.

**Response:** Well accepted and modified/ the recommendation removed as per the comment. We appreciate your comment and agree that recommendations should be based on the findings and objectives of the study.

**In the Introduction section:**

10. The backround section focuses on describing the fatality and time to death of NEC as well as its devastating consequences on healthcare outcome and costs as well. This was nice. Thus, after reading the study background, readers expect to see in the same way from your study results. However, your ‘Title’ and your study findings contrarily focused on ‘time to recovery’ rather than ‘time to death and fatality’ (Lines 60-82). I suggest re-write either of the two parts (either the background or amend your topic and findings from results through discussion and conclusion).

**Response:** Thank you for your valuable feedback regarding the alignment between the background section and the title of our study. We apologize for any confusion caused by the discrepancy between the background section and the title of our study. We are stating the absence of similar studies on the topic, making it challenging to address the concerns rose regarding the background section on the limitation part, but based on your comment, we have made efforts to amend the background section, from line 40-81.

11. The authors focused on describing the burden of NEC exhaustively. Nevertheless, they failed to state/address the main problem/focus of the study, which was time to NEC rather than the magnitude or incidence of NEC. Thus, the whole introduction section requires thourough revision in this context.

**Response:** Thank you for your comment. We appreciate your insightful comments regarding the focus and clarity of the introduction section. We apologize for the oversight in not clearly stating and addressing the main problem of the study, which is the time to necrotizing enterocolitis (NEC) rather than the magnitude or incidence of NEC. Based on your feedback, we were thoroughly revising the introduction section to ensure that it clearly highlights the main problem and focus of the study.

12. Lines 91-105: These statements can be merged with the 2nd paragraph of the introduction part since the incidence and magnitude are ways of describing diseases burden (morbidity and mortality). Avoid redundancies please!

**Response:** Thank you for your comment. We appreciate your suggestion to merge the statements in lines 91-105 with the second paragraph of the introduction to avoid redundancies. Based on your suggestion, we were carefully review the content in lines 91-105 and the second paragraph of the introduction. We removed redundancies, by merging these statements. We will make the necessary revisions to enhance the clarity and flow of the introduction section.

13. Line 109-118: “Even with early aggressive treatment, NEC still progresses and has significant morbidity with chronic gastrointestinal complications and poor neurodevelopment....”. Does it mean those adverse health outcomes related to the time of NEC recovery or the disease (NEC) itself? Rather you better state your gap statement specific to your topic of interest (time to NEC recovery) rather than NEC in general. For example, interms of longer recovery vs shorter recovery time after the diagnosis of NEC. Otherwise, your backround and statement will be suitable to study incidence of death/recovery from NEC itself rather than the timing (time of recovery). The two concepts are quite different! Please try to modify accordingly.

**Response:** Thank you for your comment and we deleted the paragraph.

**In the Methods and materials section**

14. Lines 121-22: “Institution-based retrospective follow-up study was conducted among NEC neonates in the early neonatal period...”. What does it mean early neonatal period? Does it mean you didn’t consider cases in the late neonatal period? If so you need to revise your population below and results as well. Anyways, be notified and make the required changes accordingly.

**Response:** Well accepted and all neonates who were admitted in NICU included in this study. Therefore the word early neonates deleted from the document and edited from line 84-85.

15. Line 125: “An institution-based retrospective follow-up study was conducted…” avoid redundancy. Please consider deletion.

**Response:** well accepted and it is deleted.

16. Line 140-144: I have a major concern on the appropriateness of the formula that you have used to calculate the required sample size. First, since your study is a follow-up study, single population proportion formula cannot handle/consider follow-up groups equally in the sample estimation process, and thus, is not suitable for such type of studies. Conversely, there are appropriate and scientifically sound methods of sample size determination for survival studies including Cox-model and log-rank methods among others. Why don’t you use one of these methods? Secondly, you couldn’t use a variable that you didn’t assess for sample size estimation. In your case, you used “time to reach a full enteral feeding” for sample size calculation, and you miss it in your results part though. Please try to forward if you have convincing and statistically acceptable rationale for those points!

**Response:** Dear respected reviewer, as you mentioned it is known that time to event data/study has specific sample size determination formula. But we can’t to get the required information to apply that formula during proposal preparation due to scarcity od directly related articles and assuming as time is a mean type of data we use single population mean formula to determine the sample size with using a tolerable margin of error (d=3), as you know it is possible to use a margin of error 1-5 for mean type of data. But we used tolerable margin of error (d=3) due to being conventionally agree/ think as it is neutral or not too conservative. Moreover, we used it to increase the sample size and enhance the power of the study. Related with your second concern using the variable “time to reach a full enteral feeding” for sample size calculation is due to that, the “time to reach a full enteral feeding” by definition is to mean being recovered for NEC, which is our outcome variable. So, “time to reach a full enteral feeding” was assessed in our study. It is not missed.

17. Line 157-59: “Necrotizing enterocolitis was defined as an acute disorder of the gastrointestinal in the neonatal period who presented with clinical signs or radiographic findings of NEC and who diagnosed by attending physician.” This definition is pure scientific definition and could not be considered as operational definition/measurement. Furthermore, how did you exactly diagnose NEC for the purpose of your study (Radiology findings, blood tests, clinically or what? What were the criteria used to declare whether the newborn had NEC or not? This should be clearly specified. Because sometimes cases which were diagnosed as NEC by the clinician/physicians in the charts might not fulfill the criteria of NEC diagnosis for your study (false diagnosis) whereas some others might fulfill the criteria despite the clinicians missed it actually (unrecognized cases). Therefore, please revise this section as required, and otherwise it will still be a major concern that could endanger the validity of your findings! Finally, cite the appropriate reference for the definitions provided.

**Response:** Thank you for raising important concerns regarding the definition and diagnosis of necrotizing enterocolitis (NEC) in our study. We acknowledge the importance of clearly specifying the criteria used for diagnosis and providing appropriate references. We apologize for any ambiguity in our previous description.

In our study, we defined NEC as an acute disorder of the gastrointestinal tract in the neonatal period, characterized by clinical signs or radiographic findings consistent with NEC, and diagnosed by the attending physician. We understand that this definition may not provide the level of operational detail required for research purposes.

To address your concerns and ensure the validity of our findings, we will provide a more comprehensive and specific description of the diagnostic criteria used for NEC in our study. This will include information on the specific diagnostic methods employed, such as radiology findings, blood tests, clinical assessment, or any other relevant criteria. We will also include appropriate references to support our definition and diagnostic approach.

We appreciate your valuable input, and your comments will help us improve the clarity and validity of our study. We will make the necessary revisions to address the concerns you have raised regarding the definition and diagnosis of NEC.

18. Line 173-76: “Apgar (Activity, Pulse, Grimace, Appearance and Respiration) is a quick assessment performed on a baby at 1, 5 and 10th minutes after birth and it has three levels of score: Low APGAR score 0-3 - Moderate APGAR score 4-6 - Normal APGAR score 7-10.” Please cite the source.

**Response:** Thank you for your comment and the reference is cited on line 127-130.

19. Line 197-98: “Kaplan Meier failure curve was used to estimate the time to 198 recovery during the follow-up.” Why you prefer to use the failure cureve? Time to recovery should be best expressed in the survival function rather than failure. So why you preferred the failure curve?

**Response:** Kaplan-Meier curve is actually a survival curve that estimates the probability of an event (such as recovery) occurring over time. It is commonly used to analyze time-to-event data, including time to recovery. The term "failure" in this context refers to the occurrence of the event (i.e., recovery) rather than a negative outcome. The Kaplan-Meier curve is preferred because it provides a comprehensive visualization of the probability of recovery over time for the study population. We were editing based on your comment.

20. Lines 209-210: “The Cox proportional hazard model assumption was checked for variables in the Log-log plot (graphically),...”. Log-log plot is not an assumption test, it is a model fitness check rather. Revise it please.

**Response:** Dear respected reviewer, as per our reading and learning the Log-log plot (graphically) used for Cox proportional hazard model assumption and Schoenfeld residuals test used for model fitness check. The log-log Kaplan Meier plot if parallel it fulfill Cox proportional hazard model assumption and if the plot line one cross over the other one the variable violated Cox proportional hazard model assumption.

21. Lines 209-217: I strongly advise you to remove this paragraph completely since it’s already described adequately under the ‘data processing and analysis’ section above. It is just a redundancy and avoid it except for the Cox-Snell graph!

**Response:** well accepted and the graph removed from the manuscript.

22. Lines 227-33: Could you include the reference number/research protocol number that you have received from the IRB of DBU AWHSCI here under the ‘ethical consideration section? Please state that wether the IRB waiver the study since it was retrospective study, otherwise it couldn’t be conducted from the scratch. Besides, please say something about how to ensure the confidentiality and authonomy of the data collected. What measures did you take?

**Response:** Thank you for your comment and we make arrangement on line 170.

**Results part:**

23. Table 1-6: In all tables, please add the sample size (n=…) in the table caption and specify the abbreviations and the denotation of symbols (i.e *, **) under the table footnote.

**Response:** the comment is accepted and corrected accordingly.

24. Table 4: What were the medical and surgical managements? it needs a description in the table footnote, and specify each medical management modality with their detailed weight of contribution (%) in the description above the table (Table 4).

**Response:** Thank you for your valuable comments and it is corrected accordingly: ***:** Intravenous fluid resuscitation, Antibiotics, NPO, Gastric decompression, Pain management. **: Bowel Resection, Ostomy Creation, Peritoneal Drainage, on line 200-202

25. Lines 254-56: “Most neonates in respiratory failure were assisted by respiratory support, 132 (36.6%) were assisted by intranasal oxygen (INO2) and 131 (36.3%) by Continuous Positive Airway Pressure CPAP.” What does it mean respiratory support? It need to be clearly specified/defined because respiratory support can also be provided using CPAP. So, it’s a vague term.

**Response:** thank you for your comment, we changed the phrase into type oxygen administration

26. Line 263-65: “...minimum and maximum follow-up time of 4 and 14 days... Of 361 neonates were followed the 1st neonate was recovered at the 4th day of the follow-up.” did you just consider among those recovered or among the whole samples? What was the first neonate died or transferred or censored and at what day of follow-up? You better describe minimum and maximum follow-up time in this context rather. Because, records of censored cases were considered in the statistical estimations generally. Additionally, does it mean you exclude neonates above the age of 14 days? Why did you so if it is not just a finding?

**Response:** thank you for your comment, we try to correct accordingly

a. 1st neonate who was recovered at the 4th day of the follow-up was among the whole samples (n=361)
b. We did not excluded neonates above the age of 14 days. It is the study finding, which indicate the whole 361 neonates admitted to NICU were recovered, died or referred or self-discharged within the first 14 days of follow up.
27. Lines 265-66: “The overall incidence rate of recovery was 6 per 100 (95% CI: 5.3, 6.9) person-days observation.” Why did you prefer to use a denominator of 100 for the incidence? I recommend to use a denominator of 1000 since this is the default figure. Nonetheless, if you are interested to provide the incidence per 100 person-time observations, you can convert the time scale to weeks and can estimate the incidence per 100 person-weeks rather. I recommend moderating this way.

**Response:** Well accepted and modified, which are 60 per 1000 person-days observation.

28. Lines 271-73: Don’t present everything you found here. You better pick specific time (e.g. 1st, 7th, & 14th day’s incidence). This way will be more meaningful.

**Response:** Well accepted and modified, line umber 212-213.

29. Lines 279-81: “The overall Kaplan Meire estimates identified that the probability recovery of neonates admitted with NEC was zero in the first 3 days of follow up, which relatively increase as follow up time increase.” I doubt that you interpreted your finding correctly! If the probability of recovery was zero in the first 3 days, this implies all the samples are died or censored and they have no left with any chance of survival/recovery. Because, the survival probability could not be bounced back once it reaches zero. Conversely, failure probability (chance of mortality and fatality) will be zero at the beginning of the follow-up since everyone is alive that time, and it becomes increasing with time when deaths recorded. Hence, I recommend you to re-check your survival setting before you conduct any analysis and re-run the analyses so that you will get the correct results. To sum-up, this finding and the graph above are completely wrong and need to be amended accordingly.

**Response:** Dear respected reviewer thank you for your concern, we considered that as the graph shows probability of recovery form NEC was zero in the first 3 days, which does not mean or implies all neonates are died or censored, but they may be under follow up or died/ censored. To make this decision somebody have to the patterns or flow of the graph. As you stated above in failure probability (chance of mortality and fatality) will be zero at the beginning of the follow-up since everyone is alive that time, and it becomes increasing with time when deaths recorded. Similarly, sick individuals from any illness required time to recover. Therefore the probability of recovery from NEC was zero at the first 3 days. Then as the day increase the probability of recovery from illness also increase.

30. Please delete figures 3-6 or you can provide them at the end of the manuscript as supplementary files. Log-rank tests are non-parametrics descriptive statistical methods that used to compare survival diferences among categories. But, you mixed-up it under the inferential outputs (regression outputs) which is semiparametric one. So, Log rank should be described in line with the Kaplan-Meier curve but not with the Cox-regression outputs.

**Response:** Well accepted and modified. Figures 3-6 were moved form the result section of the study to the end of the manuscript as supplementary files.

31. Table 6: Weight of the neonate contains a cell with zero record. Thus, it should not be incorporated to the Model (multivariable analysis) since it violates the test assumption (not the model assumption but test assumption). Either totally remove it or recategorize it and re-run the multivariable analysis. However, as it stands, the regression output is invalid and predictors identified cannot be acceptable.

**Response:** Well accepted, Weight of the neonates were re-categorize and analysis done again using the re-categorized result.

32. Again in Table 6: It is scientifically declared and well known that PNA, RDS and respiratory support are highly interlinked/correlated. If a neonates APGAR score is dropped below 7, it’s considered as asphyxiated and if asphyxiated you certainly suspect respiratory distress. In turn, if in respiratory distress, he/she will require respiratory support. Thereupon, you have to suspect effect modification and you don’t have to include these variables in a single model. At least you need to conduct stratified analysis (by clasifying your in two six stratum). After that, you can decide whether the association you got is statistically acceptable or invalid. Consequently, I suggest you to re-run multivariable analysis after excluding/recategorizing birth weight and removing at least two of the three inter-linked variables listed above. If you fail to do so, your finding will be completely invalid!

**Response:** Dear reviewer, thank you very much for this valuable comment and suggestions. As per you suggestion we re-analyzed the multivariable analysis after recoding the weight of the neonates and excluding the variable RDS and Respiratory support. Then, Table 6 and its’ description in the result section was modified accordingly.

**In the Discussion Part**

33. Line 333-425: Please cite the references used for the scientific justifications and explanations provided throughout the discussion section. You just cited references for comparisons, but missed to cite the justifications.

**Response:** Thank you for your feedback. The purpose of the discussion section is to interpret the results and provide an analysis of the findings in the context of existing knowledge and theories. While it is important to support comparisons and specific claims with appropriate references, citation for every justification or explanation may not be necessary based on my experience and knowledge. However, we acknowledge that the appropriate use of references can strengthen the discussion and provide readers with additional sources for further exploration. In light of your comment, the justification was based on the authors experience and knowledge.

34. Please try to make it short and precise as much as possible. As it stands, it is difficult to follow and convey the main points. It’s exhaustive and takes way interest to read as well. Hence, I recommend shrinking it to a maximum of two pages.

**Response:** Thank you for your comment and we were amend with two pages

35. Finally, please try to admit the limitation of your study that need to be taken in to consideration while using and interpreting the findings.

**Response:** Thank you for your comment and we accepted the comment

**In the Conclusions:**

36. Don’t directly repeat the results. In the conclusion, it’s expected that the major findings are interpreted and summarized meaningfully and in clinically sound manner.

**Response:** Thank you for your feedback. We agree on your comment, that the conclusion section should not merely repeat the results but should instead provide a meaningful interpretation and summary of the major findings in a clinically sound manner. In response to your comment, we were revise the conclusion section to ensure that it effectively summarizes and interprets the major findings of the study on line 306-310.

37. Please delete the statements from Line 435-455. Totally erase these from the manuscript. The conclusion should be summarized in a single paragraph succinctly and neatly.

**Response:** We accepted your concern and the content is deleted

38. Lines 456-499: Please erase all the statements from the manuscript.

**Response:** We accepted your concern and the content is deleted

39. Please provide a single statement showing the directions that need to be taken into consider addressing the identified problems (succinct recommendation statement) under the conclusion section (as the last sentence of the conclusion).

**Response:** thank you for your comment and it is directed with single sentence on line 327-329.
