# Supplementary material for: Time to recovery from necrotizing enterocolitis and its predictors among neonates admitted to Neonatal Intensive Care Unit in Bahir Dar, Ethiopia: A retrospective follow up study, 2022
Source: PLoS One. 2024 Oct 22;19(10):e0311890. doi: 10.1371/journal.pone.0311890 (PMC11495620; doi:10.1371/journal.pone.0311890)
Supplement: Attachment — Submitted filename: Review comment R2.docx [file pone.0311890.s009.docx]

**Title:** Time to Recovery from Necrotizing Enterocolitis and Its Predictors among Neonates Admitted to Neonatal Intensive Care Unitin Bahir Dar, Ethiopia: A Retrospective Follow up Study, 2022 (R2)

I would like to thank the editor for offering this opportunity to review the revised version of this work. The authors tried to partially address the comments raised in the previous review session. I appreciate the efforts devoted so far. However, still there are fundamental issues that are not yet moderated, or some unsatisfactory responses forwarded. Without addressing those issues exhaustively, the validity of the study findings will be threatened and not worth for publication.

**Major comments:**

1.1. The sample size issue remains the major concern in this manuscript. The authors could not forward statistically convincing and satisfactory justification to the previously raised concerns. It is true that single population formula can be applied if there are not studies conducted to fulfill the parameters required to apply the standard methods of sample size calculation for the given study type. However, since the authors were working on dichotomous outcome variable and categorical data, it’s completely unacceptable to use population mean. Population proportion should be the only option in such scenarios if it’s mandatory to use single population formula. How could you apply linear assumptions and parameters to estimate for binary outputs? I doubt the reproducibility of the finding since the applied sample size is one of the major factors that influence the generalizability/representativeness of the study findings.
1.2. The other thing is that “time to reach full enteral feeding” might not necessarily imply NEC recovery since NEC is not the orphan determinant of enteral feeding. I.e neonates without NEC might also encounter such problems. Inability to full enteral feeding could not be generalized to NEC since it could also be due to other causes.

2. The other major concern was that the authors could not provide clear diagnostic criteria applied to identify NEC in their study. The scientific definition provided was vague and non-specific. The diagnosis set by physicians for treatment purpose could not be directly applied for the research purpose. Rather the authors should set specific and clear criteria and differentiate NEC cases during data extraction based on the predetermined criteria (operational definition) from the charts (patient records). You declared the newborn when you got which radiorgraphic findings, which symptoms and signs? as well as how many of those criteria fulfilled?
3. Figure 1 (KM-curve): Wrong graph label and interpretation!
   3.1. The x-axis label (recovery time from NEC) is incorrect and that shoud be replaced with “analysis time”. Because the analysis time incorporates the time at risk for both recovered and censored cases (hence could not be exclusively considered as recovery time).
   3.2. Line 219-21: “The overall Kaplan Meire estimates identified that the probability recovery of neonates admitted with NEC was zero in the first 3 days of follow up…”. This statement is completely wrong. Any individual who understand English language could not interpret it as the authors intend to infer, NEVER! The authors made incorrect interpretation of the KM-graph. This is because the authors understand the statistical science, but they miss the nature of the event of interest in their own study. Researchers and statisticians need to interpret findings based on the nature of the outcome/event of interest being investigated.

**Correct interpretation of the finding in the Figure 1:** As we note from the figure (KM-curve), the survival probability (probability of recovery) remains 1 (100%) in the first 3 days, after which it slowly dropped down stepwise as the survival time increased from 0 to 14 days. This means in the first three days; every neonate has the probability to recover from NEC.

4.1. Most of the rationales stated in the discussion section of this manuscript were vague and non-specific to NEC. Majority of the explanations for the discrepancies were general views such as socio-economic, study design, setting difference, infrastructure, quality of healthcare services and so on. However, all these concepts were not declared to have direct association with NEC and the authors failed to cite the sources of such arguments if available.
4.2. Citation of references for the scientific justifications is required yet and it could not be bypassed at all. As you already stated, the purpose of the discussion section is to interpret the results and provide an analysis of the findings in the context of existing knowledge and theories. Please cite those existing knowledge and theories that could support your justification!

**Note:** Research discussion should address four major points: result interpretation, comparison, justification of discrepancies and explanation of associations, as well as implication to clinical practice. The discussion should enrich with tangible evidences and explanations so that it can be trustworthy to the scientific community. However, if it is solely written based on authors’ opinion and thoughts without tangible and citable sources, it could not be considered as scientific paper. It could be a fiction rather!

**Minor comments:**

1. Line 69-73: Please remove the first two statements of from the last paragraph of the “Introduction” section. I wonder why the authors just incorporate those statements, while it’s known that NEC is a different concept to asphyxia and RDS.
2. The authors tried to moderate the gap statement. Nevertheless, the manuscript will be benefited if the authors incorporate data related to the difference in survival rate, risk/occurrence of complications and other adverse events between longer vs shorter recovery time. For instance, neonates who recovered fast from NEC might have good prognostic outcome in terms of cognitive function, growth and development, and other long term consequences (NB: This is just an instance).
3. Line 157-58 & Line 162-63: Contradicting ideas presented yet again. Schoenfield Residuals test is an assumption (PH-assumption) test and you stated in the earlier statement as “an assumption test” and later as a “model fitness” test. Please avoid such statistical ambiguities.
4. Line 163-65: Where is the Cox-Snell residual graph? Please provide the graph and cite in “Line 165” as figure file (e.g. Fig. 1).
