# Supplementary material for: Time to recovery from necrotizing enterocolitis and its predictors among neonates admitted to Neonatal Intensive Care Unit in Bahir Dar, Ethiopia: A retrospective follow up study, 2022
Source: PLoS One. 2024 Oct 22;19(10):e0311890. doi: 10.1371/journal.pone.0311890 (PMC11495620; doi:10.1371/journal.pone.0311890)
Supplement: Attachment — Submitted filename: Response to the reviewer 2.docx [file pone.0311890.s010.docx]

Dear, I would like to thank you for offering this opportunity to review the revised version of this work. The authors tried to address the comments raised point by point.

**Comment:** Please ensure that you refer to Figure 3, 4 and 5 in your text as, if accepted; production will need this reference to link the reader to the figure.

**Response:** Thank you for the comment. I appreciate you taking the time to provide feedback. Your input is valuable and helps to improve the manuscript. I will be sure to refer to Figures 3, 4, and 5 in the text from line 264-269.
